# Taxonomic and functional heterogeneity of the gill microbiome in a symbiotic coastal mangrove lucinid species

**DOI:** 10.1038/s41396-018-0318-3

**Published:** 2018-12-05

**Authors:** Shen Jean Lim, Brenton G. Davis, Danielle E. Gill, Jillian Walton, Erika Nachman, Annette Summers Engel, Laurie C. Anderson, Barbara J. Campbell

**Affiliations:** 1 Department of Biological Sciences, Clemson University, Clemson, SC 29634-0001 USA; 2 Department of Earth and Planetary Sciences, University of Tennessee, Knoxville, TN 37996-1410 USA; 3 Department of Geology and Geological Engineering, South Dakota School of Mines and Technology, Rapid City, SD 57701-3901 USA; 4 Present Address: College of Medicine, Medical University of South Carolina, Charleston, SC 29425-8900 USA; 5 Present Address: Instituto de Medicina Tropical São Paulo, Universidade de São Paulo, São Paulo, 05403-000 Brazil

**Keywords:** Symbiosis, Symbiosis

## Abstract

Lucinidae clams harbor gammaproteobacterial thioautotrophic gill endosymbionts that are environmentally acquired. Thioautotrophic lucinid symbionts are related to metabolically similar symbionts associated with diverse marine host taxa and fall into three distinct phylogenetic clades. Most studies on the lucinid–bacteria chemosymbiosis have been done with seagrass-dwelling hosts, whose symbionts belong to the largest phylogenetic clade. In this study, we examined the taxonomy and functional repertoire of bacterial endosymbionts at an unprecedented resolution from *Phacoides pectinatus* retrieved from mangrove-lined coastal sediments, which are underrepresented in chemosymbiosis studies. The *P. pectinatus* thioautotrophic endosymbiont expressed metabolic gene variants for thioautotrophy, respiration, and nitrogen assimilation distinct from previously characterized lucinid thioautotrophic symbionts and other marine symbionts. At least two other bacterial species with different metabolisms were also consistently identified in the *P. pectinatus* gill microbiome, including a *Kistimonas*-like species and a *Spirochaeta*-like species. Bacterial transcripts involved in adhesion, growth, and virulence and mixotrophy were highly expressed, as were host-related hemoglobin and lysozyme transcripts indicative of sulfide/oxygen/CO_2_ transport and bactericidal activity. This study suggests the potential roles of *P. pectinatus* and its gill microbiome species in mangrove sediment biogeochemistry and offers insights into host and microbe metabolisms in the habitat.

## Introduction

Chemosymbiosis is widespread in marine habitats, where endosymbiotic or episymbiotic chemolithoautotrophs use inorganic chemical energy for the synthesis of organic compounds that benefit their hosts [1, 2]. One of the most ancient examples of marine chemosymbiosis is found in the bivalve family Lucinidae [3], which has a fossil record arguably dating back to the Silurian period [4]. Despite being capable of suspension feeding, all living lucinids studied to date fulfill a considerable proportion of their nutritional needs through obligate chemosymbiotic associations with gammaproteobacterial endosymbionts occupying bacteriocytes in their gills [3]. Lucinid species examined so far acquire their thioautotrophic endosymbionts from free-living environmental bacterial populations [5–9]. Enzymatic assays, stable isotope analyses, and clone-based amplicon sequencing methods demonstrate that lucinid endosymbionts mainly use energy derived from the oxidation of reduced sulfur compounds to fix inorganic carbon for their hosts [10]. Other reported functions of lucinid endosymbionts included mixotrophy, denitrification, assimilation of nitrogenous compounds, and diazotrophy [11–14].

Because of the widespread distribution of lucinids in marine habitats, ranges in host and endosymbiont phylogenetic diversity, as well as the possibility that lucinids may harbor non-thioautotrophic symbionts [15–17], the lucinid–bacteria chemosymbiotic system has the potential to address fundamental cellular to ecological questions about host–symbiont interactions, cues, and communication across individual hosts, among species, and within populations. However, there is still relatively poor understanding of lucinid and gill microbiome diversity and metabolic functions. For instance, although 16S rRNA gene sequences of thioautotrophic lucinid endosymbionts form a paraphyletic group consisting of three distinct clades [10, 18], only the genomes, transcriptomes, and proteomes of two lucinid endosymbiont species from clade A have been sequenced [13, 14]. Clade A symbionts are mainly associated with diverse seagrass-dwelling lucinids, but symbiont clades B and C are from predominately mangrove-dwelling *Anodontia* spp. and *Phacoides pectinatus*, respectively [18]. Almost no diversity or functional diversity study has centered on either of these bacterial clades.

To begin to fill these gaps, our study characterizes the metabolic repertoire of the host and gill-associated thioautotrophic bacterial endosymbiont from *P. pectinatus* Gmelin 1791 (syn = *Tellina pectinata* Gmelin 1791, *Lucina pectinata* (Gmelin 1791), *Anodontia pectinatus* (Gmelin 1791), *Lucina jamaicensis* Lamarck 1801, *Lucina funiculata* Reeve 1850). Possibly the only extant species of its genus, *P. pectinatus* possesses morphological features distinct from other lucinid bivalves, such as high levels of three types of hemoglobin in gill pigment granules, sulfur bodies, and large lysosomes [19, 20]. Molecular phylogeny studies place *P. pectinatus* as a deeply branching genus within the Lucinidae [21] and the thioautotrophic endosymbiont distant from seagrass- or other mangrove-associated lucinid endosymbionts [18, 22, 23]. This lucinid inhabits organic-rich seagrass and mangrove sediments [24] and has a widespread tropical geographic distribution that ranges from the Caribbean Sea and Gulf of Mexico to the Atlantic Ocean seaboard of South America to Brazil [25]. The unusual morphological features, phylogeny, and habitat distribution of *P. pectinatus* and its distinct thioautotrophic endosymbiont belonging to clade C have led to the hypothesis that symbiont metabolic pathways in this species are different than in other lucinid endosymbionts [8]. To test this hypothesis, we assessed gill microbiome diversity within *P. pectinatus* using 16S rRNA gene sequencing, quantitative PCR (qPCR), metagenomic sequencing, and metatranscriptomic sequencing and compared the expression profiles from *P. pectinatus* and its gill microbiome species to previously sequenced seagrass-associated lucinid endosymbiont species from clade A, including *Ca*. Thiodiazotropha endoloripes within *Loripes orbiculatus* [14] and *Ca*. Thiodiazotropha endolucinida within *Codakia orbicularis* [13].

## Materials and methods

### Sample collection

*Phacoides pectinatus* populations at Wildcat Cove, St. Lucie County, FL, USA (Figure S1), as well as their ecology, sediment geochemistry, and microbiology, have previously been investigated [23, 26] and briefly described in SI. For this study, research excursions were completed in February 2011, June 2013, July 2014, and November 2017, and live specimens were sieved from sediments hand-dug to 30 cm depth, approximately 3 m from the shoreline of *Rhizophora mangle* (red mangrove). Specimens were temporarily stored in Whirl-Pak® Bags (Nasco, Fort Atkinson, WI, USA) filled with surface water from the habitat and maintained at ambient temperature before dissection. During dissection, gill and foot tissues were separated from other body tissues. Tissues used for 16S rRNA gene sequencing and metagenomics were dissected within the same day of collection and fixed in 100% molecular-grade ethanol. Tissues used for metatranscriptomics were dissected within 30 min of collection and fixed in RNAlater. Tissues used for microscopy were fixed in 2% paraformaldehyde (pH 7) made with artificial sea water prepared using Difco™ Marine Broth 2216 formula (Becton Dickinson and Company, Franklin Lakes, NJ, USA) for 3 h at 4 °C prior to washing, sucrose infiltration, storage, hematoxylin–eosin staining, and fluorescence in situ hybridization (FISH) procedures described in Table S1 and SI. Total nucleic acids were extracted from partial gill and foot tissues using the Qiagen’s (Valencia, CA, USA) DNeasy Blood and Tissue Kit (2011 and 2013 sample collection) or Allprep DNA/RNA Mini Kit (2014 samples) after mechanical homogenization of the tissues with a motorized pestle and mortar (Argos Technologies, Elgin, IL, USA) or tissue grinder (Wheaton, Millville, NJ, USA). For further lysis, the sample was passed through a 21-gauge (0.8 mm) needle attached to a 3 mL syringe (Becton, Dickinson and Company, Franklin Lakes, NJ, USA) at least ten times and incubated at 60 °C for at least 10 min. Extracted nucleic acid concentrations were quantified fluorometrically with Qubit™ dsDNA HS and RNA assays (Life Technologies, Austin, TX, USA).

### 16S rRNA gene, metagenomic, and metatranscriptomic sequencing

From the 2014 collection, 16S rRNA gene libraries of DNA extracted from 25 *P. pectinatus* gills, cDNA from the gills of four of these individuals, and DNA from the feet of three individuals were sequenced. From the 2017 collection, libraries of DNA and cDNA extracted from three gill samples were sequenced. All libraries were sequenced on Illumina Inc’s (San Diego, CA, USA) MiSeq 2 × 250 bp platform. 16S rRNA gene library preparation procedures are described in SI. Thirteen Illumina-compatible gill metagenomic libraries and one foot metagenomic library were prepared using the Nextera DNA Sample Preparation Kit (Illumina Inc., San Diego, CA, USA) on 50 ng of DNA per sample (2011 and 2013 collection) and NEBNext® Ultra™ II DNA Library Prep Kit for Illumina® on DNA fragmented with NEBNext® dsDNA Fragmentase (New England Biolabs, Ipswich, MA, USA; 2014 collection). These libraries were sequenced on Illumina’s MiSeq 2 × 150 bp (2011 collection), 2 × 250 bp platforms (2013 collection), and HiSeq 2500 2 × 100 bp (2014 specimen) platforms. To generate long reads, metagenomic libraries were prepared from another 2014 gill specimen and two 2017 gill specimens using the Nanopore’s Rapid Sequencing Kit (Oxford Nanopore Technologies, Kidlington, Oxfordshire, UK) and sequenced on a MinIon flowcell (R9.4 nanopores) with a MinIon Mk1B sequencer. Three gill samples collected in 2014 within a 1 m^2^ quadrat were used for metatranscriptomic sequencing on Illumina’s HiSeq 4000 2 × 150 bp platform. RNAs extracted from these samples were treated with the Ambion® Turbo DNA-free™ DNase Kit (Life Technologies). Successful DNase treatment was confirmed by PCR amplification of the V9 region of bacterial 16S rRNA genes and subsequently through read-mapping analysis of sequenced metatranscriptomic libraries (SI). DNA-free RNAs were purified with the RNeasy MinElute Cleanup Kit (Qiagen). Illumina-compatible cDNA libraries were made from purified RNAs using Epicentre’s (Madison, WI, USA) Ribo-Zero rRNA removal Kit (bacteria) and ScriptSeq™ v2 RNA-Seq Library Preparation Kit, following the manufacturer’s low input protocol. The final concentration of each library was quantified with the Qubit® dsDNA HS assay (Life Technologies) and the average library insert size was determined with the Experion Automated Electrophoresis Station (Bio-Rad Laboratories, Hercules, CA, USA; 2011 and 2013 collections) and the Agilent 2100 Bioanalyzer (Agilent Technologies, Santa Clara, CA, USA; 2014 and 2017 collections). Sequencing service providers are listed in SI.

### Data analysis

Mothur v1.39.5 [27] was used for data processing for the 16S rRNA gene dataset. Operational Taxonomic Unit (OTU) clustering was performed at 99% sequence identity for higher species resolution [28] and taxonomic classification was performed against the Silva v132 database [29]. The final dataset was subsampled to the library with the smallest four-digit number size (*n* = 1269). The 16S rRNA gene analysis pipeline and qPCR procedures used to validate analysis results are documented in Table S2 and SI. Trimmed Illumina-sequenced metagenomic reads from each sequenced sample were individually assembled using IDBA-UD v1.0.9 [30]. Additionally, reads from the most complete gammaproteobacterial assembly were co-assembled with unprocessed Nanopore reads using the hybridSPAdes algorithm [31] of the SPAdes genome assembler (v3.11.1). For each assembly, contigs >1500 bp long were binned with MetaBat v0.32.4 using the ensemble binning approach [32], after read mapping with Bowtie2 v2.2.7 [33] (very sensitive local and dovetail mode) and SAMtools v0.1.19 [34]. All metagenome-assembled genomes (MAGs) were annotated with NCBI’s Prokaryotic Genome Annotation Pipeline [35]. MAGs with >90% completeness were also annotated with Rapid Annotation using Subsystem Technology (RAST) FIGfam release 70 [36]. Methods for the evaluation of sequence and MAG quality, read trimming, sequencing depth analyses, sequence comparisons with published reference genomes, and PCR validation of sequencing results are in Table S2 and SI.

Metatranscriptomic assembly and downstream analyses were performed with Trinity v2.5.1 [37]. Trimmed reads from all three metatranscriptomic libraries were co-assembled into one metatransciptome de novo with Trinity’s default parameters (*k* = 20). The co-assembly standardizes transcript IDs, lengths, and clusters across libraries for efficient downstream quantification and cross-sample comparisons [37]. Trinity’s Chrysalis module clusters transcripts with at least *k*−1 bases overlap and with sufficient reads spanning the join across both transcripts, and the Butterfly module refines the clustering and uses these transcript clusters as proxy for genes [37, 38]. Reads were mapped to the co-assembly using Bowtie2 v2.2.7’s [33] no-mixed, no-discordant, end-to-end options reporting up to 200 alignments per read (-k 200) and disallowing gaps within 1000 nucleotides of read extremes (-gbar 1000). Isoform and gene-level abundances were estimated by RNA-Seq Expectation-Maximization (RSEM) that maximizes the probability of observed variables, including read lengths, quality scores, and sequences based on RSEM’s directed graph statistical model [39]. The probability value for each isoform/gene was divided by the effective transcript/gene length, which is the average number of possible start positions of a transcript of a given length or the abundance-weighted average effective transcript lengths of a gene’s isoforms [39]. The resulting length-normalized value for each transcript/gene was divided by the sum of length-normalized values for all transcripts/genes in each sample to calculate the transcript fraction value, which was then multiplied by 10^6^ to derive the transcript per million (TPM) measure [39]. For cross-sample comparisons, TPM values were further normalized with the trimmed means of *M*-values (TMM) factor that minimizes log-fold changes across samples [40] using the edgeR Bioconductor package [41].

All assembled host and bacterial transcripts, as well as unbinned contigs from metagenomic assemblies, were annotated with Trinotate v3.1.1 (https://trinotate.github.io/), which uses the manually curated but less representative Swissprot [42] database as reference. rRNA transcripts were predicted with SortMeRNA v2 [43] using SILVA’s v119 [29] collection of archaeal, bacterial, and eukaryotic 16S rRNA, 23 S rRNA, 18 S rRNA, and 28 S rRNA gene sequences as references. Host and bacterial genes of interest were analyzed at the level of transcript clusters loosely equivalent to genes. To map transcript clusters to symbiont genes, a pan-genome for the thioautotrophic endosymbiont from *P. pectinatus*, named *Candidatus* Sedimenticola endophacoides (explained in the Results section), was created by extracting and concatenating nucleotide sequences of RAST-annotated PEGs and RNAs from six >90% complete MAGs, followed by de-duplication with CD-HIT v4.6 [44] at a global sequence identity threshold of 100%. The de-duplicated dataset was searched against the Trinity assembly using NCBI’s Basic Local Alignment Search Tool (BLAST) v2.6.0+ local blastn package [35, 45] and only the top hit was reported (-max_target_seqs 1). Similar local blastn searches were performed on other MAGs of interest for transcript cluster to gene mapping. Functions of transcript clusters of interest were inferred by comparing Trinotate’s transcript annotations with web blastp, blastn, or blastx search results [45] against the more representative NCBI’s non-redundant (nr) protein sequence or nucleotide (nt) databases [35] using the same 10^−3^ *e*-value threshold as Trinotate. For each transcript within a transcript cluster, a blastp search was performed if a likely peptide sequence was predicted by Transdecoder v5.1.0 (http://transdecoder.github.io/) based on a minimum open reading frame (ORF) length and a log-likelihood score related to the reading frame where the ORF was located. If the blastp search returned negative results or if no likely peptide sequence was predicted for a transcript, then blastn and blastx searches were performed instead. Functions of transcript clusters mapping to more than one gene were assigned based on annotations of transcript(s) within the cluster with the highest TMM-normalized TPM value(s). A transcript cluster was considered multi-mapping if more than one transcript within the cluster shared high TMM-normalized TPM values but different predicted functions and their corresponding genes were not adjacent to each other in the reference MAG.

## Results

### Site characterization

Live *P. pectinatus* had clumped distributions at Wildcat Cove in all sample years, with the highest concentrations being near the mangrove-lined coast where total organic carbon content in the sediment was highest [26]. Overall, live abundances averaged over 40 individuals per square meter [26]. Porewater dissolved sulfide and oxygen concentrations were measured and reported by Doty [26] from low-flow fluid sampling of piezometers installed near to where specimens were recovered, according to previously described methods [46]. Dissolved sulfide concentrations at Wildcat Cove (18–56 μmol/L) were an order of magnitude higher than concentrations measured from intertidal zone porewater occupied by the lucinid *Lucinoma borealis* [47]. Dissolved oxygen concentrations ranged from 78 to 125 μmol/L at quadrats adjacent to where *P. pectinatus* were collected [26].

### Gill microbiome diversity

**Fig. 1:**
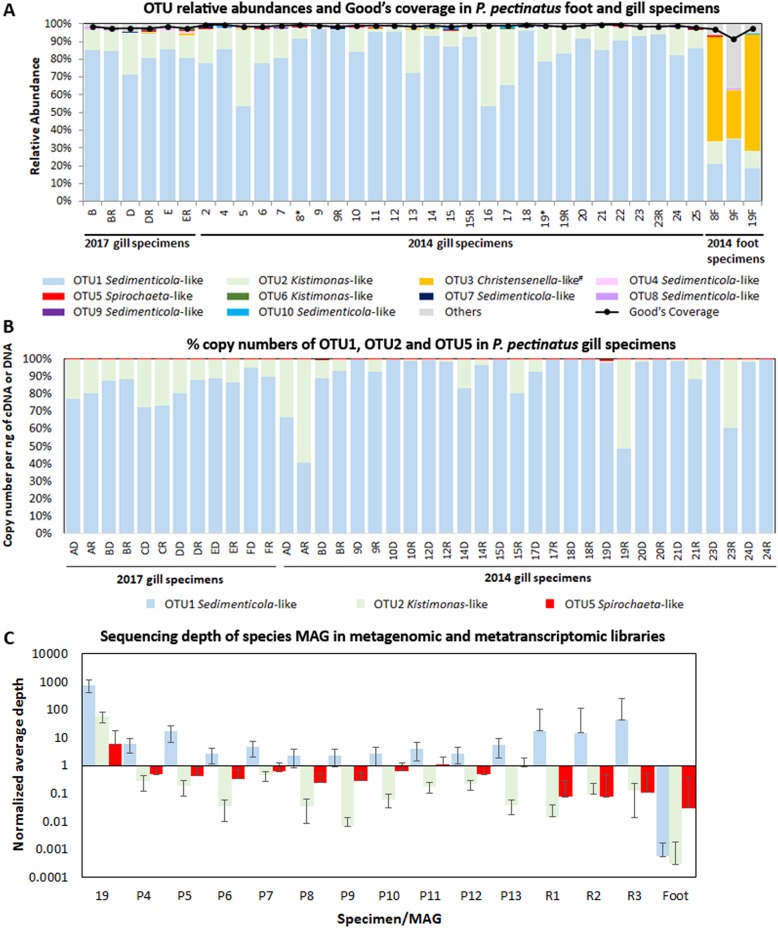
Relative abundances of **a** bacterial 16S rRNA gene OTUs and Good’s estimator of coverage [114], **b** copy numbers per ng of DNA or cDNA (%) of *Sedimenticola*-like OTU1, *Kistimonas*-like OTU2, and *Spirochaeta*-like OTU5 determined by qPCR, and **c** normalized average coverage depths with standard error bars mapped to *Ca*. Sedimenticola endophacoides, *Kistimonas*-like, and *Spirochaeta*-like MAGs  in *P. pectinatus* foot and gill specimens/libraries. “R” denotes RNA-derived cDNA specimens in **a**, **b** and metatranscriptomic libraries in **c**. Foot-associated *Christensenella*-like OTU3 indicated with “#” in **a** was classified using 0% bootstrap confidence

To examine *P. pectinatus* microbiome diversity, we sequenced 16S rRNA genes and used metagenomic and metatranscriptomic content from gill and foot samples collected in 2011, 2014, and 2017. All amplicon-sequenced DNA and cDNA samples were dominated by one gammaproteobacterial *Sedimenticola*-like species (OTU1), occurring at average 84 ± 11% relative abundance (Fig. 1a). MAGs of this species were binned from 14 separate assemblies and three co-assemblies (Table 1) and shared 100% sequence identity in the 16S rRNA gene V4 region with OTU1, as well as 99.8 ± 0.4% average nucleotide identity (ANI) and 99 ± 1% average amino acid identity (AAI; Figure S2a) with each other. These gammaproteobacterial MAGs were at least 20% smaller, and with at least 11% higher G+C content, than previously sequenced clade A thioautotrophic lucinid endosymbiont species *Ca*. Thiodiazotropha endoloripes [14] and *Ca*. Thiodiazotropha endolucinida [13] and *Sedimenticola* spp. [48, 49, 118] (Table 1). FISH using a newly designed SED642 probe targeting the 16S rRNA gene of this *Sedimenticola*-like species confirmed that the *P. pectinatus* gill bacteriocytes contained cells that matched the gammaproteobacterial MAGs (Figure S3). Results of phylogenetic analyses using 16S rRNA gene sequences (Figure S4) and ten single-copy marker genes (Figure S2b) corroborated previous reports on the distinct phylogenetic position of the thioautotrophic *P. pectinatus* endosymbiont in relation to other lucinid symbiont species [18, 22, 23]. The *Sedimenticola*-like MAGs shared 71 ± 4% ANI and 64 ± 1% AAI with sequenced clade A lucinid symbiont species, 76 ± 7% ANI and 59 ± 5% AAI with other marine thioautotrophic symbionts, and 76 ± 2% ANI and 69 ± 1% AAI to free-living *Sedimenticola* spp. [48, 49, 118] (Figure S2a). Based on the 93–95% ANI and 85–90% AAI boundaries proposed in Rodriguez-R and Konstantinidis [50], the *Sedimenticola*-like MAGs were likely a species separate from sequenced clade A lucinid symbionts, marine thioautotrophic symbionts, and *Sedimenticola* spp. Because the *Sedimenticola*-like MAGs shared the highest AAI with *Sedimenticola* spp. and the observed AAI values fall within the proposed genus boundary (55–60%) [50], we propose the name *Candidatus* Sedimenticola endophacoides for the *P. pectinatus* endosymbiont, where “endophacoides” refers the host association (“endo-” meaning “within”).

**Table 1:** Features of metagenome-assembled genomes (MAGs) assembled from *P. pectinatus* gill and foot specimens

Year of specimen collected	# reads (M)	MAG ID	Size (Mb)	No. of contigs	G+C (%)	N50 (kb)	Categorized species	CheckM^a^ completeness (%)	BUSCO^b^ completeness (%)	Contamination (%)	Strain heterogeneity (%)	MAG quality^c^
2017	2.7	N1 + P5	3.0	172	63.9	26.7	*Ca*. Sedimenticola endophacoides	96.5	95.4	1.8	25.0	High
	2.7	N1 + N3 + P5	3.0	172	63.9	26.7	*Ca*. Sedimenticola endophacoides	96.5	95.4	1.8	25.0	High
2014	114	P1	2.7	368	64.5	10.2	*Ca*. Sedimenticola endophacoides	92.3	83.8	1.2	28.6	Medium
		P2	3.3	466	52.1	8.9	*Kistimonas*-like sp.	92.9	83.8	1.5	0	Medium
		P3	1.9	177	50.0	15.1	*Spirochaeta*-like sp.	86.3	70.4	0.8	0	Medium
	2.7	N3 + P5	2.9	338	64.2	11.8	*Ca*. Sedimenticola endophacoides	96.3	95.1	1.3	0	High
2013	2.1	P4	2.8	423	64.2	8.9	*Ca*. Sedimenticola endophacoides	94.4	92.5	1.2	16.7	Medium
	2.6	P5	2.7	336	64.5	11.2	*Ca*. Sedimenticola endophacoides	95.0	93.6	1.1	0	High
	1.6	P6	1.1	440	64.0	2.5	*Ca*. Sedimenticola endophacoides	38.6	26.3	1.2	0	Low
	3.0	P7	1.8	609	64.2	3.0	*Ca*. Sedimenticola endophacoides	61.9	46.2	1.4	16.7	Medium
	1.1	P8	1.4	518	64.1	2.7	*Ca*. Sedimenticola endophacoides	52.4	35.4	1.0	0	Medium
2011	1.1	P9	0.3	150	63.0	2.0	*Ca*. Sedimenticola endophacoides	20.9	7.7	0.0	0	Low
	3.0	P10	0.5	210	62.3	2.2	*Ca*. Sedimenticola endophacoides	16.1	15.9	0.0	100.0	Low
	4.9	P11	1.2	455	62.9	2.7	*Ca*. Sedimenticola endophacoides	40.1	29.0	0.6	25.0	Low
	1.6	P12	0.4	169	63.0	2.1	*Ca*. Sedimenticola endophacoides	18.9	6.9	0.0	0	Low
	4.1	P13	1.8	585	63.3	3.3	*Ca*. Sedimenticola endophacoides	58.1	50.2	1.0	12.5	Medium
	1.2	Foot	5.8	265	37.6	2.1	Unclassified	0	0	0	0	Low

Methods for the assessment of MAG metrics and quality are described in SI

^a^Parks *et al.* [115]

^b^Simao* et al.* [116]

^c^Bowers *et al.* [117]

Besides the thioautotrophic symbiont species, we also observed lower relative abundances of a gammaproteobacterial *Kistimonas*-like OTU (average 13 ± 12%; OTU2) belonging to the order Oceanospirillales in all amplicon-sequenced DNA and cDNA samples and a *Spirochaeta*-like OTU (average 0.2 ± 0.2%; OTU5) in 25 out of the 33 gill DNA and cDNA samples (Fig. 1a). The transcriptional activity of the *Sedimenticola*-like, *Kistimonas*-like, and *Spirochaeta*-like species was confirmed by absolute qPCR quantification, where copy numbers of the OTUs in matched DNA and cDNA samples were consistent with their OTU relative abundances (Fig. 1b). Deep metagenomic sequencing of one 2014 *P. pectinatus* gill sample also binned a *Kistimonas*-like MAG (3% of reads), a *Spirochaeta*-like MAG (0.4% of reads), a *Ca*. Sedimenticola endophacoides MAG (58% of reads; Table 1), and 12 other bins with 0% completeness and no taxonomic classification. These three MAGs contained 16S rRNA gene sequences with perfect matches to their corresponding OTU sequences. Unbinned contigs comprised 89% (527,385/591,741) of all assembled contigs from this sample, out of which only 11% (59,232/527,385) had predicted protein-coding regions (SI). Reads from all sequenced metagenomic and metatranscriptomic libraries could be mapped to MAGs of the *Kistimonas*-like (0.4 ± 0.4% of MiSeq metagenomic reads and 0.1 ± 0.04% of metatranscriptomic reads) and *Spirochaeta*-like species (1 ± 0.3% of MiSeq metagenomic reads and 0.008 ± 0.003% of metatranscriptomic reads) at lower sequencing depths compared to the *Ca*. Sedimenticola endophacoides MAG (8 ± 4% of MiSeq metagenomic reads and 1 ± 0.6% of metatranscriptomic reads; Fig. 1c).

MAGs of *Ca*. Sedimenticola endophacoides, the *Kistimonas*-like species, and the *Spirochaeta*-like species shared <70% ANI and <56% AAI with each other (Figure S2a). Phylogenetic analyses using 16S rRNA gene sequences clustered the *Kistimonas*-like OTU sequences with potentially pathogenic *K. scapharcae* from a dead ark clam *Anadara broughtonii* [51], skin-associated *K. asteriae* from the starfish *Asterias amurensis* [52], and gill-associated Oceanospirillales from the limid bivalve *Acesta excavata* [53] (Figure S4). The *Spirochaeta*-like OTU sequence was most closely related to spirochete endosymbionts in the gutless marine worm *Olavius* [54, 55] and loosely associated with spirochete sequences retrieved from a *L. kazani*-like lucinid [56] (Figure S4). Genomic sequences of these closest relatives of both species are not yet available in public databases. Foot microbiome diversity in *P. pectinatus* is described in SI.

### Metagenomic and metatranscriptomic analyses

Sequenced gill cDNA libraries showed consistent read coverages of the co-assembled metatranscriptome and pairwise Pearson correlations of >0.8 across replicates (Figure S5). A total of 1,563,787 transcripts were assembled, out of which 85% (average length 364 ± 262 bp) were without protein-coding region and functional annotation (SI). In all, 11% of the total transcripts (average length 989 ± 1181 bp) could be mapped to gene/protein homologs. These were grouped into 91,465 transcript clusters (loosely equivalent to genes), from which a subset (3%) mapped to the bacterial MAGs of interest. As such, it should be noted that the quality of gene/transcript annotations is heavily dependent on the completeness of the MAGs and the reference databases used. Although we made every effort to search for absent genes and pathways in the unbinned gill metagenomes, incompletely binned MAGs used to make inferences may still contain missing genes and functions. The lack of host genomic data and the high abundances of unclassifiable sequences in the gill metagenomes and metatranscriptomes imply that functional analyses can be skewed toward annotated genes/transcripts that would overlook novel genes [57]. Also, gene/transcript annotations based on homology may not be accurate predictors of reaction mechanisms [57] and even function (in the case of novel paralogs). Transcript quantification can also be influenced by swift changes in mRNA expression occurring between sample collection and fixation, as well as mRNA turnover that causes rapidly degrading mRNAs to exhibit inaccurately low transcripts per million (TPM) values.

### Host-related functions

**Fig. 2:**
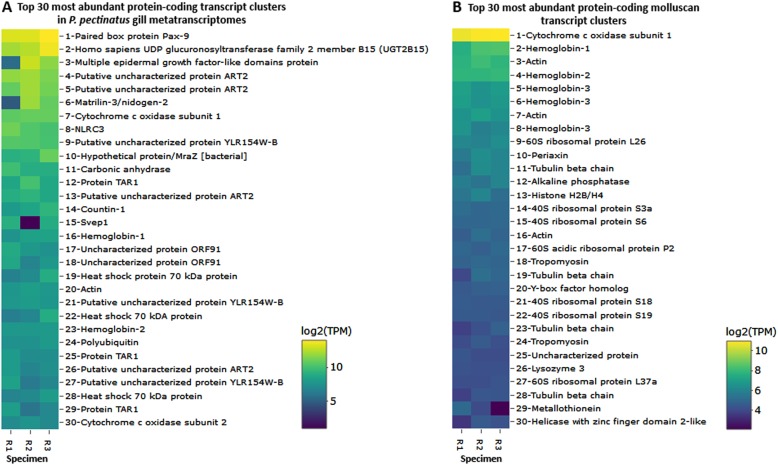
Log_2_-transformed TMM-normalized TPM of gene products of the 30 most abundantly expressed protein-coding transcript clusters **a** mapped to any species and **b** mapped to the phylum Mollusca in sequenced *P.* *pectinatus* gill metatranscriptomes (specimens R1, R2, and R3). UDP uridine diphosphate, ORF open reading frame

Host-related rRNA gene transcript clusters made up two thirds of the 30 most abundantly expressed transcripts in the gill metatranscriptomes (Figure S6). Highly expressed eukaryotic and/or molluscan protein-coding genes included those encoding the respiratory cytochrome c oxidase subunits, hemoglobins 1 and 2, and actin (Fig. 2). A carbonic anhydrase transcript cluster related to the mangrove killifish (*Kryptolebias marmoratus*) was the 11th most abundantly expressed in the gill metatranscriptome (average 696 ± 260 TPM; Fig. 2a), while another molluscan transcript cluster encoding for a nacrein-like protein with putative carbonic anhydrase function [58] was expressed in only 1 out of the 3 specimens at 0.1 TPM. The top 30 most abundant molluscan transcript clusters also included transcripts encoding hemoglobin 3 (average 104 ± 25 TPM), ribosomal proteins (average 32 ± 18 TPM), other cytoskeletal proteins (tubulin and tropomyosin; average 33 ± 19 TPM), and lysozyme 3 (average 19 ± 20 TPM; Fig. 2b). Similarly, transcript clusters matching gene ontology [59] terms relevant to hemoglobin, cytoskeletal, and ribosomal functions were among the most abundant in the phylum Mollusca (Figure S7). Transcript clusters involved in the defense response to bacteria (GO:0042742; Figure S7) were potentially relevant to symbiosis. These included molluscan transcript clusters encoding lysozyme 1 (average 6 ± 4 TPM), lysozyme 3, an antibacterial glycoprotein aplysianin-A [60]/muscosal glycoprotein achacin [61] (average 7 ± 2 TPM), the H_2_O_2_-generating flavoenzyme L-amino oxidase [62] (average 7 ± 1 TPM), and nitric oxide synthase (average 0.6 ± 1 TPM).

### Endosymbiont functions

**Fig. 3:**
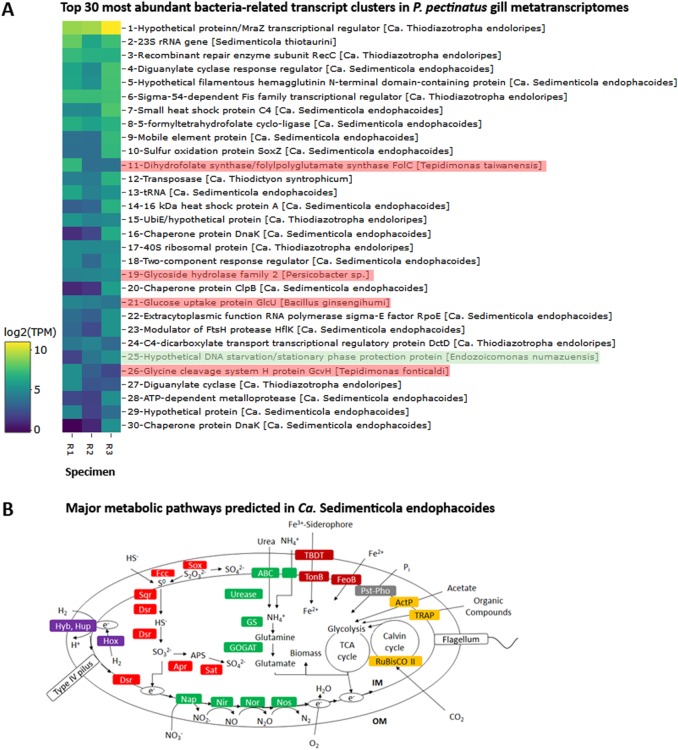
Log_2_-transformed TMM-normalized TPM of gene products of **a** the 30 most abundantly expressed protein-coding transcript clusters mapped to species (square brackets) from the domain Bacteria and **b** morphological features and major metabolic pathways predicted in *Ca*. Sedimenticola endophacoides. In **b**, the transcript cluster mapped to a non-thioautotrophic gammaproteobacterial species (*Endozoicomonas numazuensis*) is highlighted in green while transcript clusters mapped to non-gammaproteobacterial taxa are highlighted in pink. UbiE ubiquinone/menaquinone biosynthesis C-methyltransferase, FtsH ATP-dependent zinc metalloprotease, Hyb membrane bound [Ni-Fe] hydrogenase 2, Hup uptake hydrogenase, Hox soluble NAD-dependent hydrogenase, S^0^ elemental sulfur, Fcc flavocytochrome c-sulfide dehydrogenase, Sqr sulfide:quinone oxidoreductase, Sox sulfur oxidation enzyme complex, Dsr reverse dissimilatory sulfite reductase enzyme system, Apr adenylylsulfate reductase, APS adenosine-5’-phosphosulfate, Sat sulfate adenylyltransferase, ABC ATP-binding cassette transporters, GS glutamine synthetase, GOGAT glutamine oxoglutarate aminotransferase (glutamate synthase), Nap periplasmic dissimilatory nitrate reductase, Nir cytochrome nitrite reductase *cd*_1_, Nor nitric oxide reductase, Nos nitrous oxide reductase, TBDT TonB-dependent transporter, TonB TonB-ExbB-ExbD complex, FeoB ferrous iron transport protein, Pst phosphate specific transport, Pho phosphate regulon, PolyP polyphosphate granule, ActP acetate permease, TRAP tripartite ATP-independent periplasmic transport, RuBisCO ribulose-1,5-bisphosphate carboxylase/oxygenase, TCA cycle tricarboxylic acid cycle, IM inner membrane, OM outer membrane

**Fig. 4:**
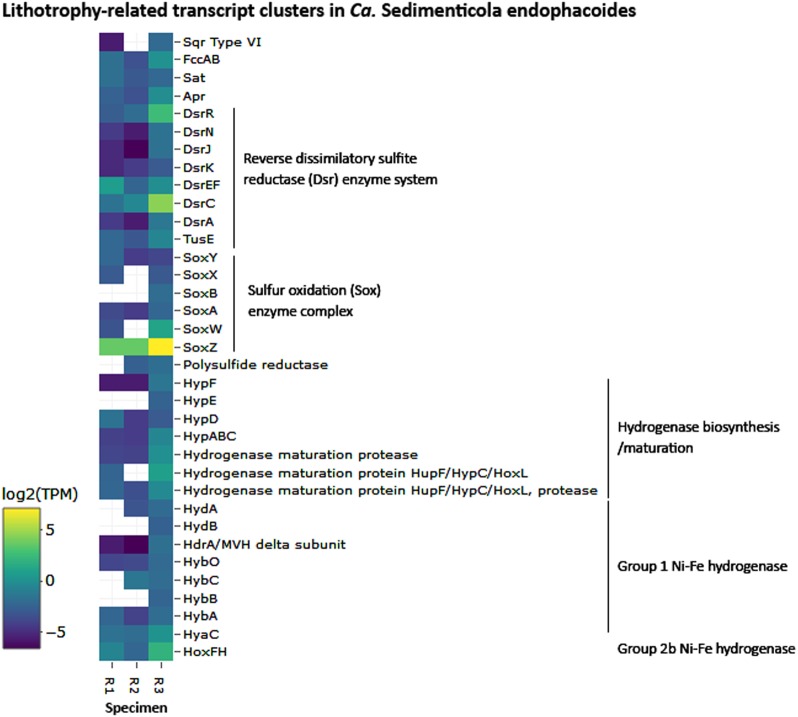
Log_2_-transformed TMM-normalized TPM of gene products of lithotrophy-related transcript clusters mapped to *Ca*. Sedimenticola endophacoides. Transcript clusters with zero TPM values are represented as white cells. Sqr sulfide:quinone oxidoreductase, Fcc flavocytochrome c-sulfide, Sat sulfate adenylyltransferase, Apr adenylylsulfate reductase, Tus sulfur carrier proteins homologous to some Dsr proteins, Hyp hydrogenase pleiotropy operon involved in the biosynthesis and maturation of [Ni-Fe] hydrogenases, Hup regulatory uptake hydrogenase, Hox soluble NAD-dependent hydrogenase, Hyd periplasmic Ni-Fe hydrogenase, HdrA/MVH heterodisulfide reductase/methylviologen reducing hydrogenase, Hyb membrane-bound Ni-Fe hydrogenase 2, HyaC membrane-bound Ni-Fe-hydrogenase I cytochrome b subunit

**Fig. 5:**
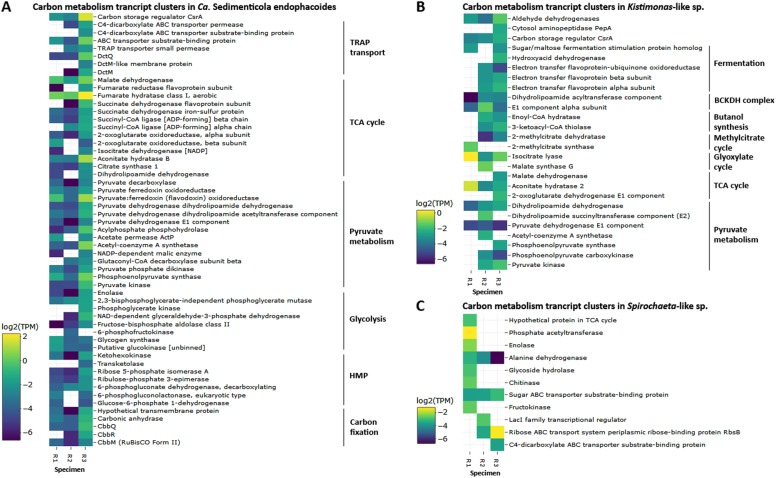
Log_2_-transformed TMM-normalized TPM of transcript clusters encoding gene products involved in carbon metabolism mapped to **a** *Ca*. Sedimenticola endophacoides, **b** the *Kistimonas*-like species, and **c** the *Spirochaeta*-like species. Transcript clusters with zero TPM values are represented as white cells. TRAP tripartite ATP-independent periplasmic transport, TCA cycle tricarboxylic acid cycle, HMP hexose monophosphate shunt, Dct dicarboxylate transport proteins, Cbb proteins encoded by the Calvin–Bassham–Benson cycle operon, BCKDH complex branched-chain alpha-keto acid dehydrogenase complex, LacI lactose operon repressor

Sixteen of the 30 most abundant bacteria-related transcript clusters could be mapped to *Ca*. Sedimenticola endophacoides, while 8 mapped to the species’ relatives (Fig. 3a). Many of these were housekeeping and stress response genes (Fig. 3 and SI). *Candidatus* Sedimenticola endophacoides expressed lithoautotrophic genes involved in sulfur oxidation, hydrogen oxidation, and carbon fixation (Figs. 3–5). Transcript clusters involved in thiotrophic sulfur oxidation (*sox*) and reverse dissimilatory sulfite reductase enzyme system-adenylylsulfate reductase-sulfate adenylyltransferase (*dsr*-*apr*-*sat*) pathways [63, 64] were detected in the transcriptome at TPM values between 0.07 (DsrK) and 55 (SoxZ; Figs. 3–4). Variants of sulfide:quinone oxidoreductase (Sqr), hydrogenases, and ribulose-1,5-bisphosphate carboxylase/oxygenase (RuBisCO) genes utilized by chemosynthetic marine symbionts differed across lineages (Table 2), and *Ca*. Sedimenticola endophacoides expressed a unique combination of type VI Sqr (average 0.09 ± 0.1 TPM), group 1 membrane-bound (average 0.2 ± 0.2 TPM) and group 2b soluble NAD-dependent (average 2 ± 2 TPM) Ni-Fe hydrogenases, and type II RuBisCO (average 0.08 ± 0.06 TPM) genes (Figs. 4 and 5). Expressed heterotrophy-related genes included those involved in dicarboxylate transport (average 0.2 ± 0.3 TPM) and a complete tricarboxylic acid (TCA) cycle (average 0.4 ± 0.8 TPM; Fig. 5a). *Candidatus* Sedimenticola endophacoides is capable of respiration on oxygen and nitrogenous compounds (average 0.2 ± 0.4 TPM; Fig. 3b). However, compared to other chemosynthetic marine symbionts that utilize a variety of terminal oxidases for aerobic respiration, we only detected genes and transcript clusters encoding subunits for the cbb3-type terminal oxidase (average 0.4 ± 0.5 TPM) in *Ca*. Sedimenticola endophacoides (Table 2). *Candidatus* Thiodiazotropha spp. are capable of nitrogen fixation and assimilatory nitrate and nitrite reduction [13, 14], and relevant transcripts mapped to *Ca*. Thiodiazotropha endoloripes, but not *Ca*. Sedimenticola endophacoides, were identified in the gill metatranscriptomes at average 0.3 ± 0.3 TPM (Table S4 and SI). Key genes in these pathways were, however, not detected in the sequenced *P. pectinatus* gill metagenomes (Tables S4–S5 and SI), suggesting that the transcripts were rare. MAGs of *Ca*. Sedimenticola endophacides encoded and expressed genes for urease and the urease accessory protein UreE (0.09 ± 0.1 TPM), urea ABC transporter (0.2 ± 0.2 TPM), and ammonium transporter (average 0.06 ± 0.1 TPM; Figure S8). Transcripts involved in type I, II, and possibly type III and VI, secretion systems were also observed in this species (Figures S9 and SI). Particularly, like *Ca*. Thiodiazotropha spp., *Ca*. Sedimenticola endophacoides may utilize the type I secretion system [65] potentially for the secretion of hemolysin A (average 1 ± 1 TPM), colicin V (average 0.4 ± 0.4 TPM), and repeats in toxin (average 0.2 ± 0.03 TPM; Figure S9). A transcript cluster encoding a hypothetical filamentous hemagglutinin N-terminal domain-containing iron-responsive protein (average 104 ± 80 TPM) secreted by the two-partner secretion system [66] was also the fifth most abundant in the bacterial metatranscriptomes (Fig. 3a). Genes to combat H_2_O_2_ stress, including those encoding the hydrogen peroxide-inducible genes activator (average 0.3 ± 0.3 TPM), superoxide dismutase (average 0.05 ± 0.06 TPM), and an alkyl hydroperoxide reductase subunit C-like protein (average 0.7 ± 0.8 TPM) were also expressed in *Ca*. Sedimenticola endophacoides. Other genetic functions in *Ca*. Sedimenticola endophacoides are presented in Table S6 and SI.

**Table 2:** Comparison of genomic features among *Ca*. Sedimenticola endophacoides, free-living *Sedimenticola*spp. [48, 118], and bacterial symbionts, including clade A thioautotrophic lucinid symbionts the lucinid thioautotrophic symbiont clade A [13, 14], a vesicomyid clam gill symbiont (*Ca*. Ruthia magnifica) [119], a solemyid clam gill symbiont [97], bathymodiolin mussel gill symbionts (*Bathymodiolus septemdierum* and *Bathymodiolus thermophilus*) [75, 120], deep-sea tubeworm trophosomal symbionts (*Ridgeia piscesae*, *Riftia pachyptila,* and *Tevnia jerichonana*) [93, 121], a marine nematode ectosymbiont (*Ca.* Thiosymbion oneisti) [14], and a deep-sea scaly-foot snail esophageal gland symbiont [98]. ‘+’ denotes a feature annotated in a genome, whereas ‘−' denotes a feature not yet sequenced in a genome

Function	*Ca*. Sedimenticola endophacoides	*S. thiotaurini*	*S. selenatireducens*	*Ca*. Thio-diazotropha endoloripes	*Ca*. Thio-diazotropha endolucinida	*Ca*. Ruthia magnifica	*Solemya velum s*ymbiont	*Bathymodiolus* spp. symbionts	Deep-sea tubeworm symbionts	*Ca*. Thiosymbion oneisti	*Crysomallon squamiferum* symbiont
Sulfur oxidation	Sqr type VI	Sqr type I and VI			Sqr type I, III, and VI	Sqr type I	Sqr type I	Sqr type I and VI			Sqr type I
Hydrogen oxidation	Group 1 and 2b (NAD-dependent) Ni-Fe hydrogenase			Group 1 and 2a Ni-Fe hydrogenase		−	Group 1 and 2b (NAD-dependent) Ni-Fe hydrogenase	Group 1 and 2a Ni-Fe hydrogenase		−	Group 1 and 2a Ni-Fe hydrogenase
Carbon fixation	RuBisCo II		RuBisCO Iaq and II	RuBisCo Iaq	RuBisCo Iaq and II	RuBisCO II	RuBisCO Iaq	RuBisCO Iaq	RuBisCO II	RuBisCO Iaq	RuBisCO Iaq and II
Nitrogen fixation	−	+	−	+	+	−	−	−	−	+	−
Assimilatory nitrate and nitrite reduction	−	−	−	+	−	−	−	+	+	+	+
Urea hydrolysis	+	+	−	+	−	−	+	−	−	+	−
Oxygen respiration	cbb3-type terminal oxidase	cbb3, aa3, and cytochrome d ubiquinol oxidases		cbb3- and aa3-type terminal oxidases							cbb3, aa3, and cytochrome d ubiquinol oxidases

Sqr sulfide:quinone oxidoreductase, RuBisCO ribulose-1,5-bisphosphate carboxylase/oxygenase

### Other gill microbiome functions

**Fig. 6:**
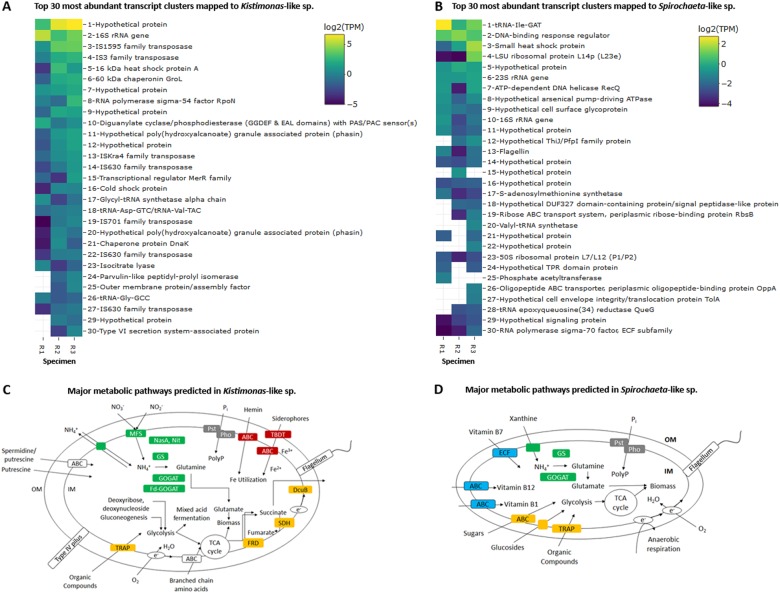
Log_2_-transformed TMM-normalized TPM of gene products of the 30 most abundantly expressed protein-coding transcript clusters mapped to **a** the *Kistimonas*-like species and **b** the *Spirochaeta*-like species and major metabolic pathways predicted in **c** the *Kistimonas*-like species and **d** the *Spirochaeta*-like species. Transcript clusters with zero TPM values in **a**, **b** are represented as white cells. MFS major facilitator superfamily transporter, Nas assimilatory nitrate reductase, Nit assimilatory nitrite reductase, GS glutamine synthetase, GOGAT glutamine oxoglutarate aminotransferase (glutamate synthase), Fd-GOGAT ferrodoxin-dependent glutamate synthase, Pst phosphate-specific transport, Pho phosphate regulon, PolyP polyphosphate granule, TBDT TonB-dependent transporter, ABC ATP-binding cassette transporters, DcuB C4-dicarboxylate uptake family transporter, SDH succinate dehydrogenase, FRD fumarate reductase, TCA cycle tricarboxylic acid cycle, TRAP tripartite ATP-independent periplasmic transport, ECF energy-coupling factor transporter

Highly expressed protein-coding transcript clusters homologous to protein sequences from other non-thioautotrophic bacterial taxa, including *Tepidimonas* spp., *Persicobacter* sp., and *Bacillus ginsengihumi*, were also observed in the gill metatranscriptomes (Fig. 3a). A transcript cluster encoding a hypothetical DNA starvation/stationary phase protection protein from *Endozoicomonas numazuensis*, a relative of the *Kistimonas*-like species, was also identified (Fig. 3a). Seven of the 30 most abundant transcript clusters mapped to the *Kistimonas*-like species encoded transposases (average 3 ± 4 TPM; Fig. 6a). Two transcript clusters encoding poly(hydroxyalcanoate) granule-associated protein (phasin) involved in the fermentative synthesis of polyhydroxyalkanoate storage granules [67] were also highly expressed in the species (average 1 ± 1 TPM; Fig. 6a). Heterotrophy-related genes associated with other fermentation processes were expressed by the species at lower average TPM values of 0.09 ± 0.08, along with TCA cycle genes (average 0.2 ± 0.3 TPM; Fig. 5b and Fig. 6c). Transcript clusters linked to fatty acid catabolism and synthesis, including those involved in the glyoxylate cycle (average 0.4 ± 0.5 TPM) [68], methylcitrate cycle (average 0.08 ± 0.2 TPM) [69, 70], and the branched-chain alpha-keto acid dehydrogenase complex (BCKDH complex; average 0.1 ± 0.1 TPM) [71, 72], were also observed (Fig. 5b). A transcript cluster encoding a type VI secretion system-associated protein (average 0.5 ± 0.5 TPM) was among the most abundant in the species’ transcriptomes (Fig. 6a). The *Kistimonas*-like species likely respires aerobically with both cbb3-type cytochrome c oxidase (average 0.07 ± 0.07 TPM) and cytochrome bd ubiquinol oxidase (average 0.2 ± 0.3 TPM). For nitrogen assimilation (Fig. 6c), only two genes encoding NAD(P)H-dependent assimilatory nitrite reductase (average 0.02 ± 0.03 TPM; consistent with PCR results in SI) and type I glutamine synthetase (average 0.06 ± 0.1 TPM) were expressed in the species.

The most abundant transcript clusters mapped to the ~78% complete MAG of the lower abundance *Spirochaeta*-like species encoded transporters for ribose (average 0.2 ± 0.2 TPM) and oligopeptide (average 0.1 ± 0.2 TPM; Fig. 6b). Besides ribose, the species could potentially utilize other carbon sources through transcripts encoding sugar ABC transporter substrate-binding protein (average 0.09 ± 0.02 TPM), chitinase (average 0.06 ± 0.1 TPM), glycoside hydrolase (average 0.05 ± 0.08 TPM), and C4-dicarboxylate ABC transporter substrate-binding protein (average 0.05 ± 0.08 TPM; Fig. 5c). Other genetic functions in the *Kistimonas*-like species and *Spirochaeta*-like species are presented in Fig. 6, Tables S6 and SI.

## Discussion

Systems-level approaches utilizing next-generation sequencing technologies successfully reveal host–microbe and microbe–microbe interactions in different invertebrate symbioses [73–76] but have not been widely applied to lucinid–bacteria chemosymbioses. Currently, the lack of genomic, transcriptomic, and proteomic data for lucinids hosting gammaproteobacterial clades B and C thioautotrophic endosymbionts results in a poor understanding of the metabolism, inter- and intra-species diversity, and molecular interactions between these partners that may impact their surrounding coastal habitat and other organisms in the environment. In this study, we focused on describing the gill microbiomes of the mangrove-dwelling *P. pectinatus* that hosts the poorly characterized clade C lucinid endosymbiont species. This is the first investigation to describe the functional repertoire of (1) a lucinid symbiont species belonging to clade C, (2) a lucinid clam, and (3) other bacterial species in a lucinid gill microbiome. Our comparative genomics analyses showed thioautotrophy, respiration, and nitrogen assimilation metabolic differences among the clade C *P. pectinatus* endosymbionts, clade A lucinid symbionts, and other thioautotrophic marine symbionts, while host transcriptomes revealed candidate genes putatively involved in symbiont/microbiome selection, regulation, and nutrient transfer. Metagenomic and metatranscriptomic analyses also uncovered consistency among members of the gill microbiome, including a *Kistimonas*-like species and a *Spirochaeta*-like species that have previously been associated with a variety of marine invertebrates but not yet been comprehensively studied in lucinid clams. Additional insights into the lucinid-bacteria chemosymbiosis is now possible, and these findings may help in species conservation, habitat management [77–79], and even in fisheries productivity [80], which are areas of ongoing research.

Compared to previously sequenced lucinid clade A endosymbiont species and other thioautotrophic symbionts, *Ca*. Sedimenticola endophacoides encoded a unique combination of low-affinity type VI Sqr that functions best at high sulfide concentrations [81, 82], form II RuBisCO that is less efficient at discriminating between oxygen and CO_2_ [83], and the high affinity cbb3-type terminal oxidase that performs best at low oxygen concentrations [84]. These genomic differences suggest that *Ca*. Sedimenticola endophacoides experiences a more oxygen-poor extracellular and/or intracellular environment compared to *Ca*. Thiodiazotropha spp. Although pore water sulfide concentrations at Wildcat Cove were higher than previous studies [47], pore water dissolved oxygen concentrations were similar to those from sub-tropical coastal mangroves [85] and seagrass rhizomes [86] that have the potential to harbor lucinids. Sulfide and oxygen levels in the clam gills are likely regulated through hemoglobins, which can be partially saturated with oxygen [87]. As such, sulfide-reactive hemoglobin 1, which has a higher oxygen dissociation rate than oxygen-reactive hemoglobins 2 and 3, may be confined to the symbiotic mollusc gills [88]. In support of previous literature, we observed high expression levels of host-related hemoglobin 1, 2, and 3 genes responsible for sulfide  and oxygen transport [88–90]. Despite genomic evidence for the maintenance of low intracellular oxygen that would be conducive for nitrogen fixation, which can contribute to the lucinid’s diet and seagrass health [13, 14, 91], *Ca*. Sedimenticola endophacoides, unlike *Ca.* Thiodiazotropha spp., is likely incapable of diazotrophy. In lieu of nitrogen fixation, we speculate that *Ca*. Sedimenticola endophacoides may utilize urea and ammonium as its nitrogen source because these transcripts were detected.

Expression levels of autotrophy-related transcripts encoding RuBisCO and Calvin cycle enzymes in relation to other transcripts were much lower for *Ca*. Sedimenticola endophacoides than previously reported in *Ca*. Thiodiazotropha endoloripes [14] and other symbiotic bivalve species that expressed RuBisCO form Iaq [75, 92], where these transcripts were among the most abundant in the transcriptomes. Low RuBisCO protein levels (~1%) were similarly observed in the tubeworm *Riftia pachyptila* thioautotrophic symbiont, which was discovered to produce proteins involved in an additional oxygen-sensitive reductive TCA cycle [93–95]. Although *Ca*. Sedimenticola endophacoides expressed genes encoding 2-oxoglutarate oxidoreductase that may reverse the 2-oxoglutarate to succinyl-CoA step in the TCA cycle, we did not identify any gene for citrate lyase or citryl–coenzyme A synthetase subunit that potentially converts citrate to oxaloacetate or acetate [93, 95]. Mixotrophy has previously been inferred in *Ca*. Thiodiazotropha endoloripes [14], as well as thioautotrophic symbionts in a variety of other marine organisms [96–98], and is a likely possibility for *Ca*. Sedimenticola endophacoides because of encoded and expressed genes associated with the dicarboxylate transport and TCA cycle, as well as the correlation of *P. pectinatus* live abundances to sediment organic carbon content [26]. However, gene expression and geochemical data are insufficient support for proven mixotrophy, and more carbon assimilation experiments will be needed to determine such mechanisms in *Ca*. Sedimenticola endophacoides.

Besides *Ca*. Sedimenticola endophacoides, we also identified genes and transcripts belonging to other bacterial taxa in the *P. pectinatus* gill metagenomes and metatranscriptomes. Transcripts mapped to *Ca*. Thiodiazotropha endoloripes were noted in the gill metatransciptomes and could originate from unbinned contigs in the gill metagenomes or closely related species co-occurring in the gill microbial population. In all sequenced gill samples, we observed the consistent presence of a *Kistimonas*-like species related to the metabolically versatile Oceanospirillales species that can be symbiotic [99–102], parasitic [103], or pathogenic [51, 104]. In bivalves, parasitic Oceanospirillales have been identified from nuclei in the vent mussel *Bathymodiolus* spp. [103]. Another Oceanospirillales species with unknown functions was also reported in gills from *A. excavata* [53]. Consistent with previous genomic reports on Oceanospirillales species, we observed high expression of various families of transposases in the *Kistimonas*-like species, which may facilitate rapid   adaptation to new hosts or environments [105–107]. We also identified lower relative abundances of a *Spirochaeta*-like species in most gill samples, as well as transcriptional evidence of their activity. Spirochete species have been associated with a *L. kazani*-like lucinid [56], the symbiotic gutless oligochete worm *Olavius* [55, 108], and episymbionts of the hydrothermal vent worm *Alvinella pompejana* [109].

Metatranscriptomic analyses showed that these three bacterial species may utilize distinct carbon sources. Specifically, *Ca*. Sedimenticola endophacoides may participate in mixotrophy in addition to thioautotrophy, whereas the *Kistimonas*-like species performs fermentation and fatty acid catabolism, and the *Spirochaeta*-like species breaks down chitin, sugars, and dicarboxylate compounds. To identify cellular locations of the *Kistimonas*-like and the *Spirochaeta*-like species within the host gill tissue, we designed multiple FISH probes targeting various 16S rRNA gene regions of the *Kistimonas*-like and *Spirochaeta*-like species, as these species showed positive DNA and cDNA amplification from gill specimens. However, in contrast to positive FISH signals for *Ca*. Sedimenticola endophacoides, we repeatedly failed to get unambiguous true positive signals for the *Kistimonas*-like and *Spirochaeta*-like species. This could be because of the low abundances of these species within the tissue samples, the hybridization efficiency of the designed probes, the resolution of the confocal microscopy, and/or other technical issues. Without microscopic data, we are unable to determine the location of these species and entirely rule out that they could be environmental contaminants, transient gill-filtered bacteria,   pathogens, or parasites. More sensitive techniques, such as catalyzed reporter deposition–FISH [110] and hybridization chain reaction [111], should be performed to validate the presence of these bacteria species in the gills of *P. pectinatus*.

Our gill metatranscriptomic analyses also revealed potential host–microbiota interactions involved in the establishment and maintenance of lucinid–bacteria relationships. In *P. pectinatus*, transfer of nutrients, including carbon and possibly B vitamins and cofactors, from symbiont to host may predominantly occur via host lysosomal digestion. The high abundances of host-associated lysozyme-encoding transcripts observed in this study may indicate the presence of active lysosomes, supporting previous reports of lysosomes in the host gills [19] and in the vent mussel *Bathymodiolus azoricus* [75]. We speculate that host selection may include the secretion of bactericidal lysozyme and other compounds (SI), which can be countered by gill microbiome species. Presumably to decrease competition from closely related species/strains, as speculated in the *Eupyrmna*–*Vibrio* symbiosis [112], *Ca*. Sedimenticola endophacoides encoded and expressed genes for the production and secretion of bactericidal colicin [113], which were also annotated in the *Kistimonas*-like species MAG. A strongly expressed transcript cluster encoding a hypothetical filamentous hemagglutinin N-terminal domain-containing iron-responsive protein responsible for adhesion to host tissues [66] was also observed in *Ca*. Sedimenticola endophacoides, while fatty acid synthesis and catabolism-related genes encoding isocitrate lyase, BCKDH, and proteins within the methylcitrate cycle in *Kistimonas*-like species have been attributed to growth and virulence in other bacterial taxa [68–72]. Other genes associated with virulence and bacterial secretion systems were also detected in the genomes and transcriptomes of *Ca*. Sedimenticola endophacoides. However, their significance in the lucinid–bacteria chemosymbiosis is unclear. Nevertheless, the speculated roles of bactericidal, adhesion, and virulence compounds would have to be tested using experimental approaches to better understand host selection and microbiome persistence.

Overall, this study provides insight into the metabolic functions and interactions of *P. pectinatus*, its thioautotrophic symbiont, and other gill microbiome species. Our discovery of distinct metabolic differences between the clade C endosymbiont, clade A lucinid symbionts, and other marine thioautotrophic symbionts, as well as the consistent presence and activity of other bacterial taxa in the gills, suggests that lucinid gill microbiome diversity is currently underrepresented in the literature and should warrant more investigative efforts, including additional host–microbiome meta-omics, imaging, and experimental studies. It is well established that the lucinid gill microbiome and their interactions with the host and/or the environment contribute to nutrient cycles in coastal marine sediments; however, many details have been lacking. Our metagenomic and metatranscriptomic analyses of mangrove-associated lucinid host and gill microbiome functions provide a systems biology perspective of host and microbiome physiology that is relevant to host–microbe and microbe–microbe interactions.

## Electronic supplementary material

Figure S1

Figure S2

Figure S3

Figure S4

Figure S5

Figure S6

Figure S7

Figure S8

Figure S9

Table S1

Table S2

Table S3

Table S4

Table S5

Table S6

Supplementary Information
