# Correction: Taxonomic and functional heterogeneity of the gill microbiome in a symbiotic coastal mangrove lucinid species

**DOI:** 10.1038/s41396-020-0585-7

**Published:** 2020-01-15

**Authors:** Shen Jean Lim, Brenton G. Davis, Danielle E. Gill, Jillian Walton, Erika Nachman, Annette Summers Engel, Laurie C. Anderson, Barbara J. Campbell

**Affiliations:** 1 Department of Biological Sciences, Clemson University, Clemson, SC 29634-0001 USA; 2 Department of Earth and Planetary Sciences, University of Tennessee, Knoxville, TN 37996-1410 USA; 3 Department of Geology and Geological Engineering, South Dakota School of Mines and Technology, Rapid City, SD 57701-3901 USA; 4 Present Address: College of Medicine, Medical University of South Carolina, Charleston, SC 29425-8900 USA; 5 Present Address: Instituto de Medicina Tropical São Paulo, Universidade de São Paulo, São Paulo, 05403-000 Brazil

**Keywords:** Symbiosis, Symbiosis

**Correction to: The ISME Journal**

10.1038/s41396-018-0318-3

Since publication of the original paper, the authors noticed that a typesetting error meant Table 2 was typeset incorrectly. The correctly laid out table is displayed below. The HTML and PDF versions of this paper have also been updated. The publisher wish to apologise for any inconvenience caused.

**Table 2:** Comparison of genomic features among *Ca*. Sedimenticola endophacoides, free-living *Sedimenticola* spp. (Flood et al. 2015; Louie et al. 2016), and bacterial symbionts, including the lucinid thioautotrophic symbiont clade A (Petersen et al. 2016; Konig et al. 2016), a vesicomyid clam gill symbiont (*Ca*. Ruthia Magnifica; Roeselers et al. 2010), a solemyid clam gill symbiont (Dmytrenko et al. 2014), bathymodiolin mussel gill symbionts (*Bathymodiolus septemdierum* and *Bathymodiolus thermophilus*; Ikuta et al. 2016; Ponnudurai et al. 2017), deep-sea tubeworm trophosomal symbionts (*Ridgeia piscesae*, *Riftia pachyptila* and *Tevnia jerichonana*; Gardebrecht et al. 2012; Perez and Juniper 2016), a marine nematode ectosymbiont (*Ca*. Thiosymbion oneisti; Petersen et al. 2016), and a deep-sea scaly-foot snail esophageal gland symbiont (Nakagawa et al. 2014).

Function	*Ca*. Sedimenticola endophacoides	*S. thio-taurini*	*S. selenati-reducens*	*Ca*. Thio-diazotropha endoloripes	*Ca*. Thio-diazotropha endolucinida	*Ca*. Ruthia magnifica	*Solemya velum s*ymbiont	*Bathymodiolus* spp. symbionts	Deep-sea tubeworm symbionts	*Ca*. Thiosymbion oneisti	*Crysomallon squamiferum* symbiont
Sulfur oxidation	Sqr type VI	Sqr types I and VI			Sqr types I, III and VI	Sqr type I	Sqr type I	Sqr types I and VI			Sqr type I
Hydrogen oxidation	Groups 1 and 2b (NAD-dependent) Ni-Fe hydrogenase			Groups 1 and 2a Ni-Fe hydrogenase		−	Groups 1 and 2b (NAD-dependent) Ni-Fe hydrogenase	Groups 1 and 2a Ni-Fe hydrogenase		−	Groups 1 and 2a Ni-Fe hydrogenase
Carbon fixation	RuBisCo II		RuBisCO Iaq and II	RuBisCo Iaq	RuBisCo Iaq and II	RuBisCO II	RuBisCO Iaq	RuBisCO Iaq	RuBisCO II	RuBisCO Iaq	RuBisCO Iaq and II
Nitrogen fixation	−	+	−	+	+	−	−	−	−	+	−
Assimilatory nitrate and nitrite reduction	−	−	−	+	−	−	−	+	+	+	+
Urea hydrolysis	+	+	−	+	−	−	+	−	−	+	−
Oxygen respiration	cbb3 type terminal oxidase	cbb3, aa3 and cytochrome d ubiquinol oxidases		cbb3 and aa3 type terminal oxidases							cbb3, aa3 and cytochrome d ubiquinol oxidases

‘+’ denotes a feature annotated in a genome while ‘−’ denotes a feature not yet sequenced in a genome

*Sqr* sulfide:quinone oxidoreductase, *RuBisCO* ribulose-1,5-bisphosphate carboxylase/oxygenase

