# Supplementary figures and images for: Taxonomic and functional heterogeneity of the gill microbiome in a symbiotic coastal mangrove lucinid species
Source: ISME J. 2018 Dec 5;13(4):902–20. doi: 10.1038/s41396-018-0318-3 (PMC6461927; doi:10.1038/s41396-018-0318-3)

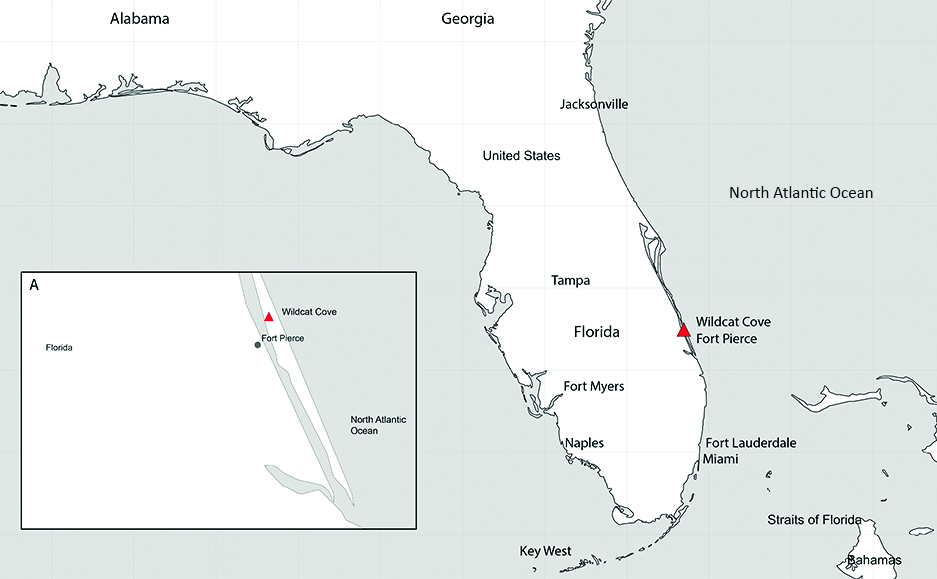

Supplement: Supplementary file 1 — Figure S1 [file 41396_2018_318_MOESM1_ESM.tif]

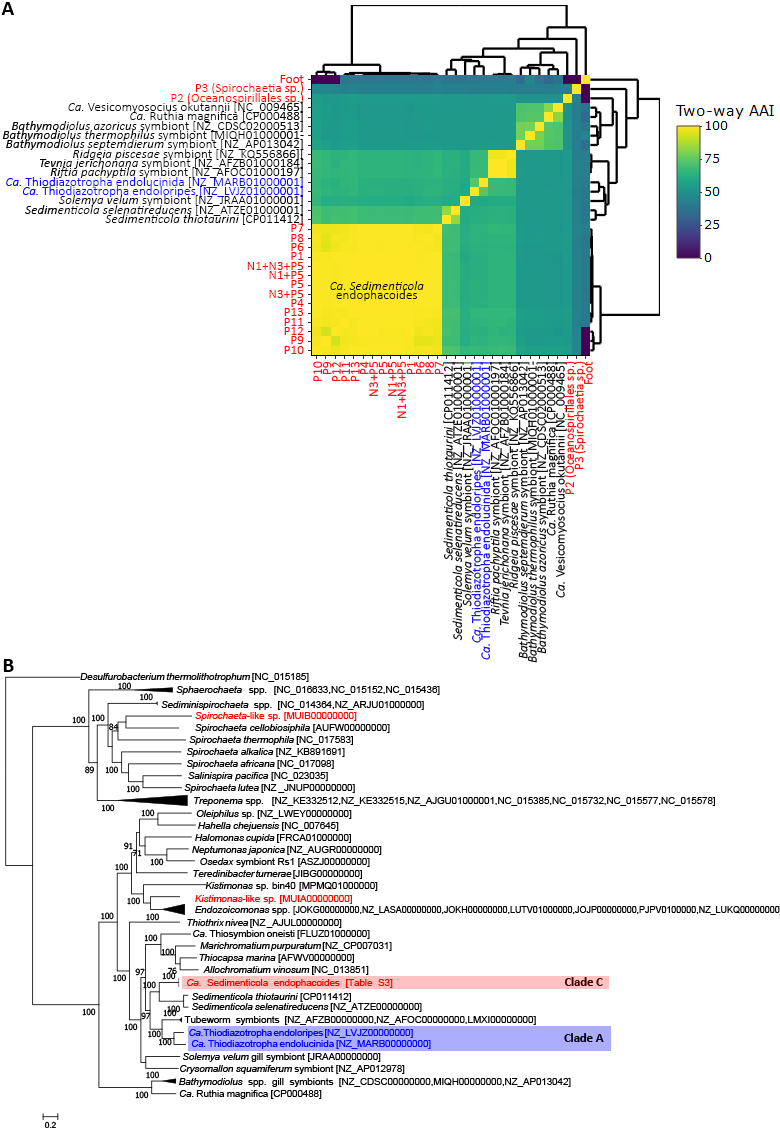

Supplement: Supplementary file 2 — Figure S2 [file 41396_2018_318_MOESM2_ESM.tif]

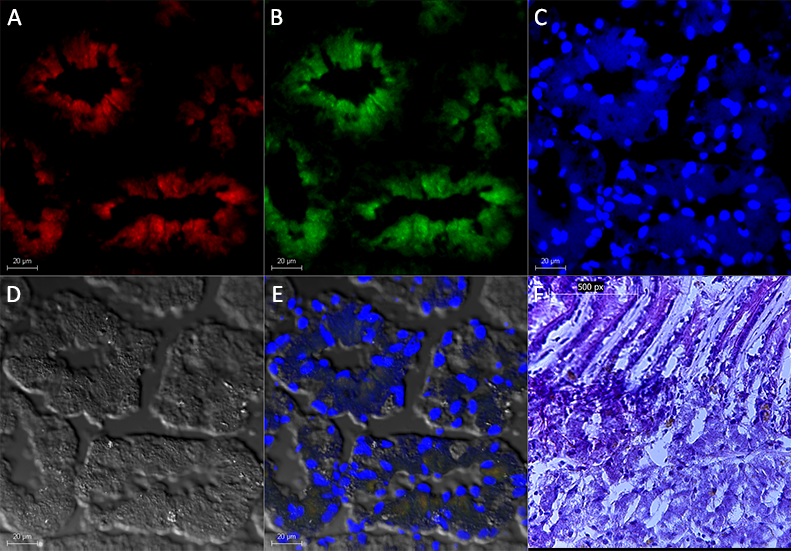

Supplement: Supplementary file 3 — Figure S3 [file 41396_2018_318_MOESM3_ESM.tif]

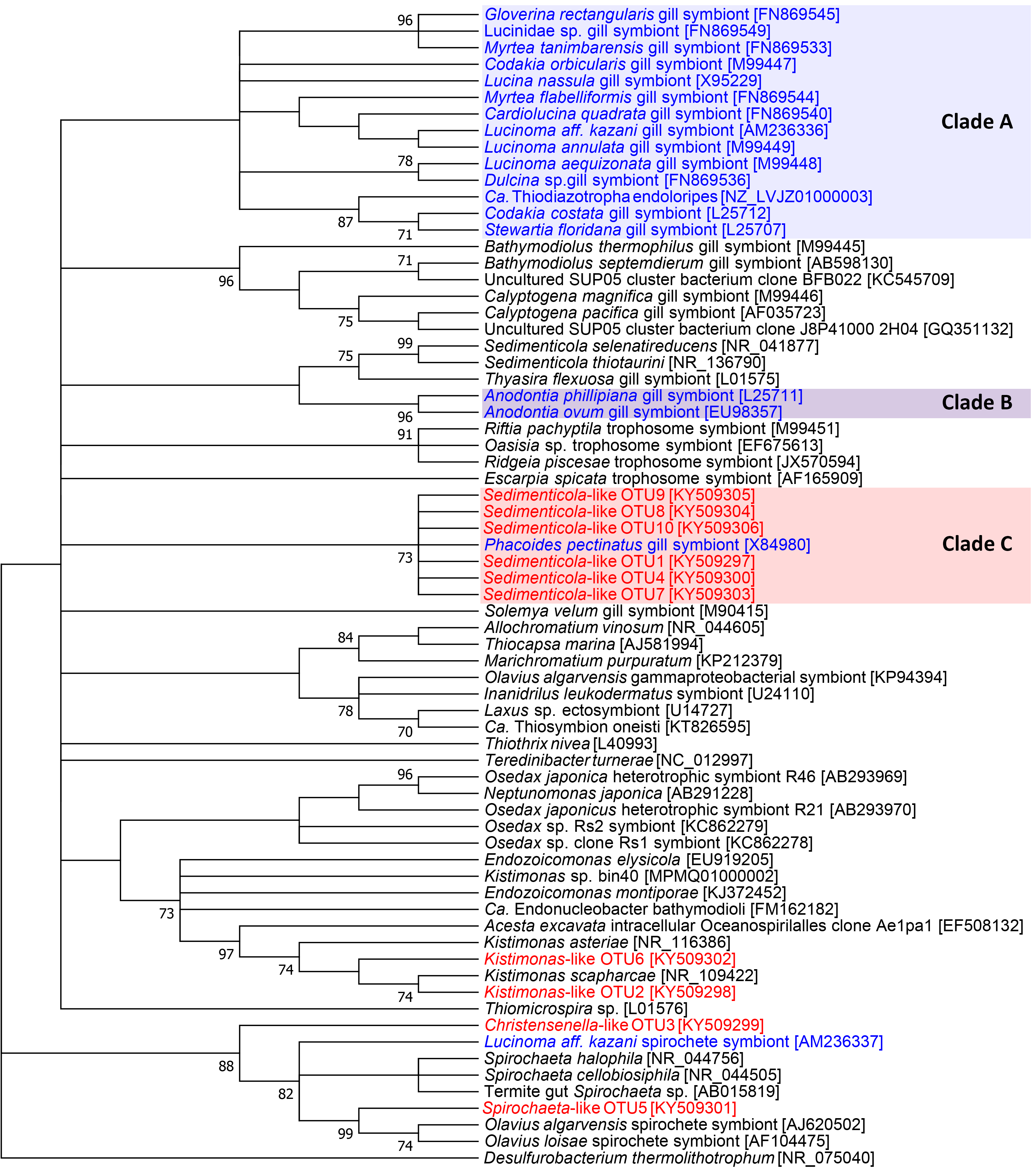

Supplement: Supplementary file 4 — Figure S4 [file 41396_2018_318_MOESM4_ESM.tif]

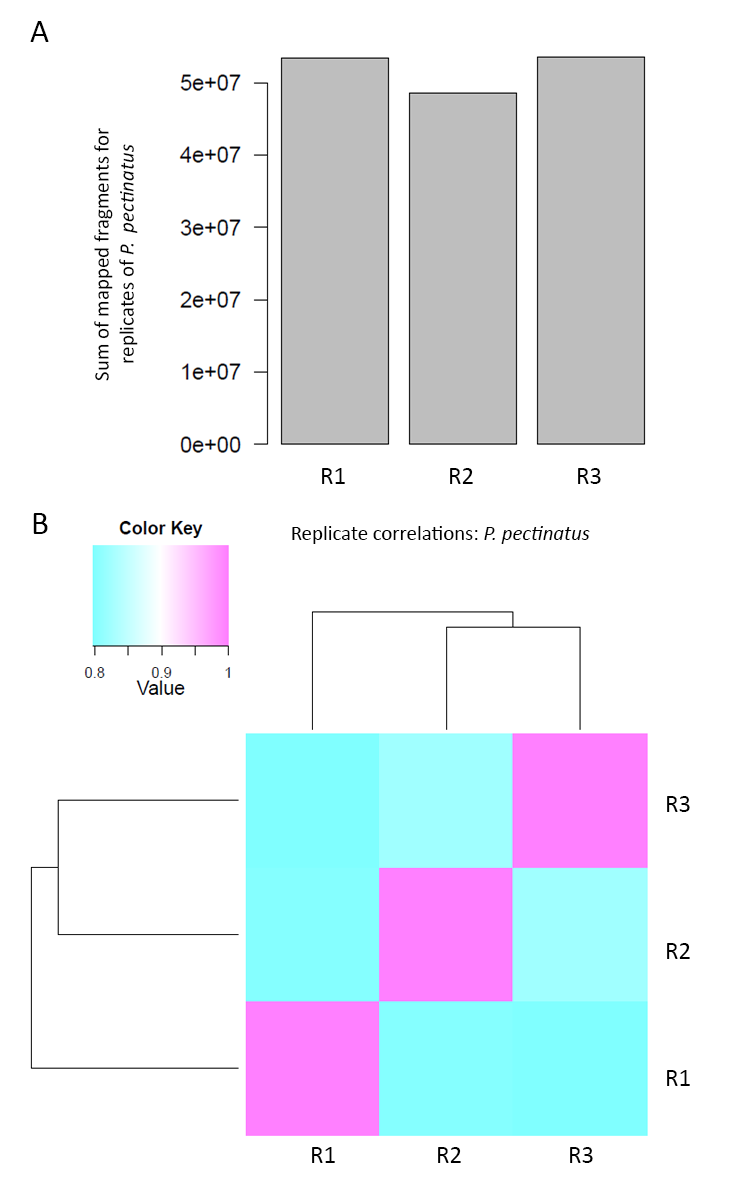

Supplement: Supplementary file 5 — Figure S5 [file 41396_2018_318_MOESM5_ESM.tif]

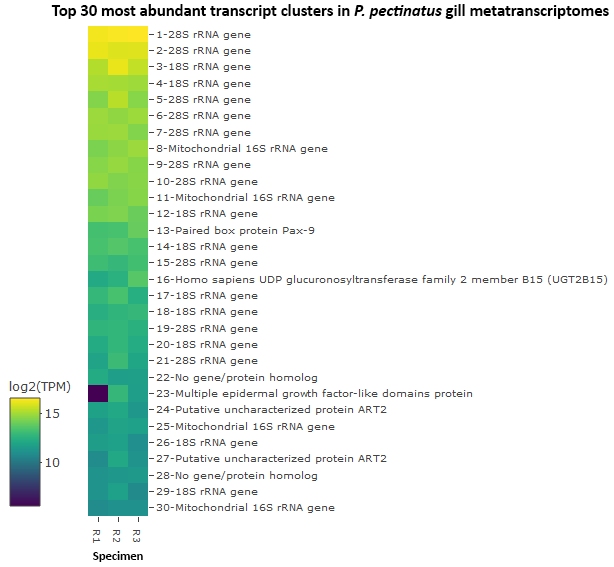

Supplement: Supplementary file 6 — Figure S6 [file 41396_2018_318_MOESM6_ESM.tif]

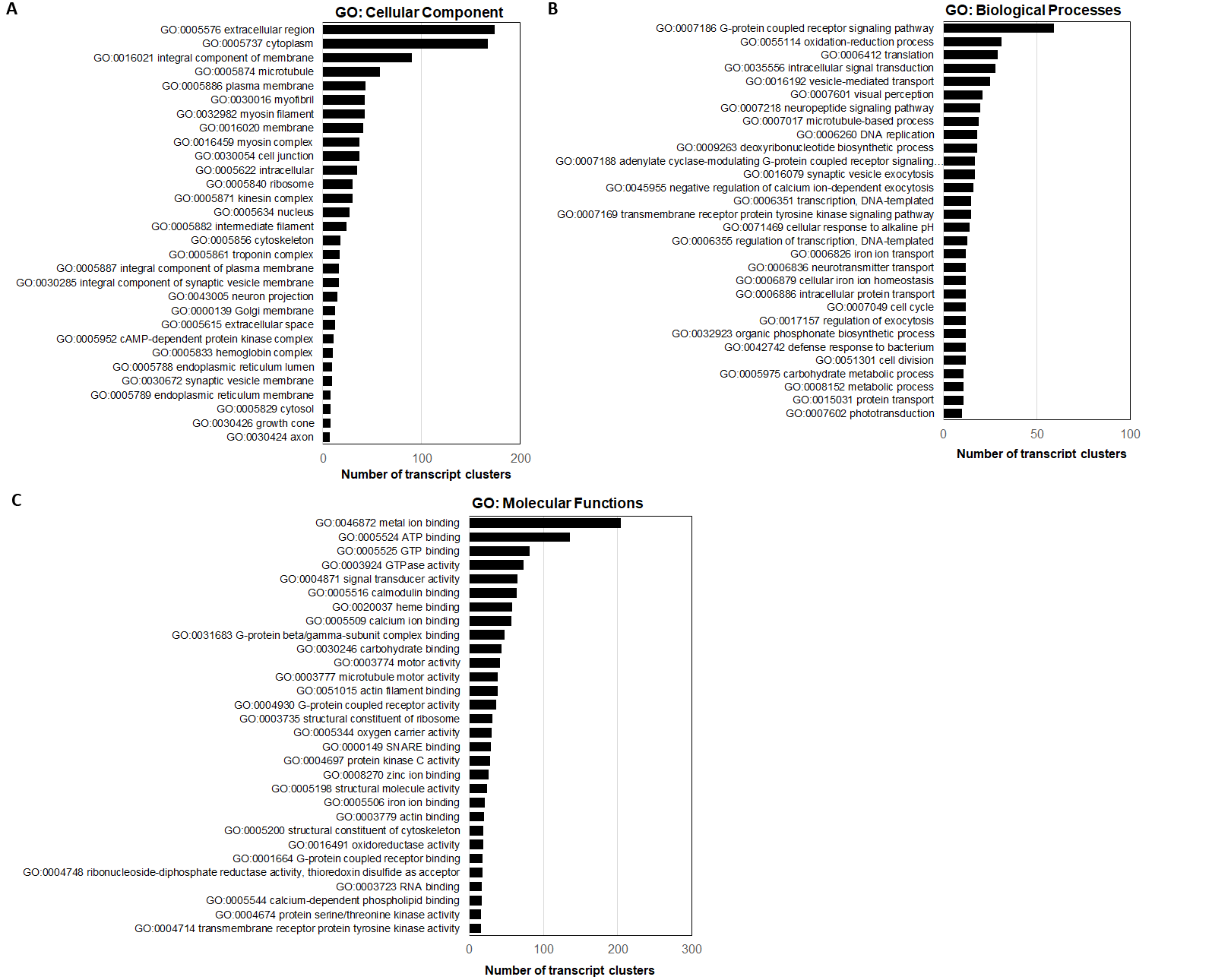

Supplement: Supplementary file 7 — Figure S7 [file 41396_2018_318_MOESM7_ESM.tif]

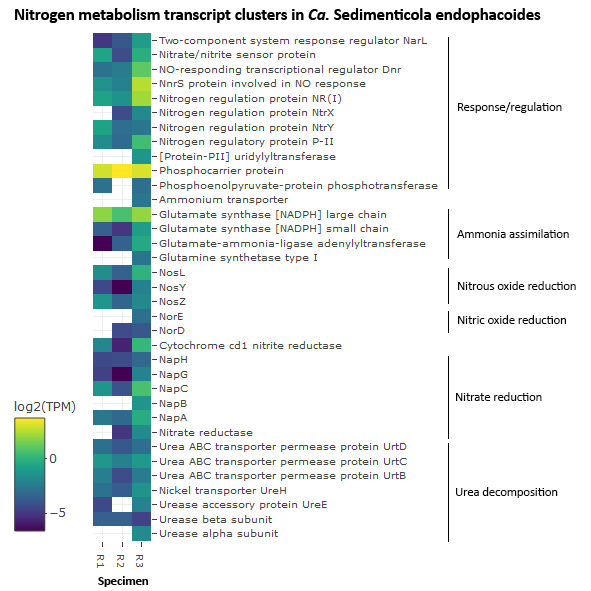

Supplement: Supplementary file 8 — Figure S8 [file 41396_2018_318_MOESM8_ESM.tif]

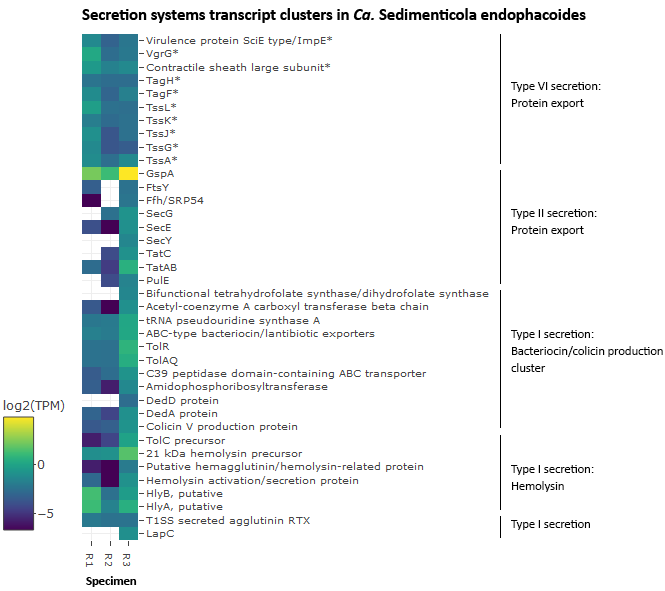

Supplement: Supplementary file 9 — Figure S9 [file 41396_2018_318_MOESM9_ESM.tif]
