# Supplementary material for: Taxonomic and functional heterogeneity of the gill microbiome in a symbiotic coastal mangrove lucinid species
Source: ISME J. 2018 Dec 5;13(4):902–20. doi: 10.1038/s41396-018-0318-3 (PMC6461927; doi:10.1038/s41396-018-0318-3)
Supplement: Supplementary file 10 — Table S1 [file 41396_2018_318_MOESM10_ESM.docx]

**Table S1.** Sequence comparisons of the *Ca.* Sedimenticola endophacoides SED642 fluorescence in situ hybridization (FISH) probe designed in this study with 16S rRNA gene sequences of other related bacterial species obtained from NCBI's 16S ribosomal RNA sequence database (NCBI Resource Coordinators, 2016).

| **Probe and 16S rRNA gene target** | **Sequence (5’ 🡪 3’)** |
| --- | --- |
| SED642 probe for *Ca*. Sedimenticola endophacoides (this study) | ACCATACTCTAGCCTGCCAG |
| BangT-642 probe for thiotrophic symbiont of *Bathymodiolus* sp. (Duperron *et al.*, 2005) | CCT----------T------ |
| *Salinispirillum* *marinum* GCWy1 (NR_134169)^a^ | -------------------- |
| *Methylophaga* *nitratireducenticrescens* JAM1 (NR_074321) | -------------------- |
| *Marinomonas* *arenicola* KMM 3893 (NR_112826) | -------------------- |
| *Marinomonas* *rhizomae* IVIA-Po-145 (NR_116233) | -------------------- |
| *Marinomonas* *arctica* 328 (NR_043882) | -------------------- |
| *Methylophaga* *alcalica* M39 (NR_028824) | -------------------- |
| *Pseudomonas* *amygdali* AL1 (NR_036999) | -------------------- |
| *Methylosphaera* *hansonii* AM6 (NR_026033) | -------------------- |
| *Burkholderia* *singularis* LMG 28154 (NR_152632), other *Burkolderia* strains^b^ | X------------------- |
| *Paraburkholderia* *caffeinilytica* strain CF1 (NR_152088) and other *Paraburkholderia* strains | X-------------C----- |
| *Colwellia meonggei* MA1-3 16S (NR_133732), and other *Umboniibacter* and *Solobacterium* strains | XXX----------------- |
| *Oceanospirillum beijerinckii* subsp. *pelagicum* IFO 13612 (NR_112017), and other *Oceanospirillum*, *Oceanobacter*, and *Vibrio* strains | XXXX---------------- |
| *Yimella* *radicis* py1292 (NR_152030), and other *Yimella*, *Calidifontibacter*, and *Neisseria* strains | ------------------XX |
| *Pseudomonas* *cerasi* 58 (NR_146827) and other *Pseudomonas, Methyloparacoccus* strains | -------------U------ |
| *Methylocaldum* *marinum* S8 (NR_126189) and other *Methylocaldum*, *Marinobacter*, and *Endothiovibrio* strains | ----C--------------- |
| *Thiohalomonas* *denitrificans* HLD 2 (NR_044097) | ----A--------------- |
| *Methylohalobius* *crimeensis* 10Ki (NR_042198) | ----G--------------- |
| *Thioalkalispira* *microaerophila* ALEN 1 (NR_025239), misc. *Pseudomonas* strains | ------------U------- |

^a^ -, identical to probe sequence

^b^X, no base pair reported
