# Supplementary material for: Taxonomic and functional heterogeneity of the gill microbiome in a symbiotic coastal mangrove lucinid species
Source: ISME J. 2018 Dec 5;13(4):902–20. doi: 10.1038/s41396-018-0318-3 (PMC6461927; doi:10.1038/s41396-018-0318-3)
Supplement: Supplementary file 11 — Table S2 [file 41396_2018_318_MOESM11_ESM.docx]

**Table S2.** List of PCR and qPCR primers used in this study.

| **Primer** | **Sequence (5’->3’)** | **Annealing temperature** | **Reference** |
| --- | --- | --- | --- |
| Universal 16S rRNA gene primer 1369F | CGGTGAATACGTTCYCGG | 53°C | Suzuki *et al.*, 2000 |
| Universal 16S rRNA gene primer 1492R | GGWTACCTTGTTACGACTT | 53°C | Suzuki *et al.*, 2000 |
| Universal M13 forward (-21) primer | GTAAAACGACGGCCAG | 55°C | NA |
| Universal M13 reverse primer | CAGGAAACAGCTATGAC | 55°C | NA |
| Universal 16S rRNA gene primer 27F | AGAGTTTGATCMTGGCTCAG | 55.8°C | Lane, 1991 |
| Universal 16S rRNA gene primer 1391R | GACGGGCGGTGTGTRCA | 55.8°C | Turner *et al.*, 1999 |
| *Sedimenticola*-like OTU1 1417F | AGCTAATACCGCATACGCCC | 56.3°C | This study |
| *Sedimenticola*-like OTU1 1580R | GTGTCTCAGTCCCAGTGTGG | 56.3°C | This study |
| *Kistimonas-*like OTU2 90F | CCTGGGAACTGCATCCCAAA | 57°C | This study |
| *Kistimonas-*like OTU2 231R | GCACCTCAGCGTCAGTGTTA | 57°C | This study |
| *Spirochaeta*-like OTU5 15F | GCGTTGTTCGGAATTATTGGGC | 56°C | This study |
| *Spirochaeta*-like OTU5 226R | TCAGCGTCAATCTTTGGCCA | 56°C | This study |
