# Supplementary material for: Taxonomic and functional heterogeneity of the gill microbiome in a symbiotic coastal mangrove lucinid species
Source: ISME J. 2018 Dec 5;13(4):902–20. doi: 10.1038/s41396-018-0318-3 (PMC6461927; doi:10.1038/s41396-018-0318-3)
Supplement: Supplementary file 12 — Table S3 [file 41396_2018_318_MOESM12_ESM.docx]

**Table S3.** NCBI accession numbers of raw read and sequence data generated in this study. All data are linked to NCBI’s BioProject ID PRJNA368737 (NCBI Resource Coordinators, 2016).

| **Database** | **Accession numbers** | **Dataset description** |
| --- | --- | --- |
| Sequence Read Archive (SRA) | SRR5381359-SRR5381390 | 16S rRNA gene sequence reads (V4 region) from the 2014 collection |
|  | SRR6473966-SRR6473965 | 16S rRNA gene sequence reads (V4 region) from the 2017 collection |
|  | SRR5381472-SRR5381483 | Paired-end metagenomic reads sequenced using various Illumina platforms |
|  | SRR6472705-SRR6472704 | Metagenomic reads sequenced using the Oxford Nanopore MinION sequencing platform |
|  | SRR6473829-SRR6473827 | Metatranscriptomic reads sequenced using the Illumina HiSeq 4000 platform |
| GenBank | KY509297-KY509306 | Nucleotide sequences of OTU1 to OTU10 |
|  | MUHZ00000000-MUIM00000000 | Draft genomic assemblies from Illumina-sequenced reads |
|  | PQCO00000000-PQCQ00000000 | Draft hybrid genomic assemblies from Illumina and Oxford Nanopore reads |
