# Supplementary material for: Taxonomic and functional heterogeneity of the gill microbiome in a symbiotic coastal mangrove lucinid species
Source: ISME J. 2018 Dec 5;13(4):902–20. doi: 10.1038/s41396-018-0318-3 (PMC6461927; doi:10.1038/s41396-018-0318-3)
Supplement: Supplementary file 13 — Table S4 [file 41396_2018_318_MOESM13_ESM.docx]

**Table S4.** Summary of nitrogen fixation (*nif*) transcripts identified in the gill metatranscriptomes of *P. pectinatus*. The closest protein homolog and organism of each transcript were determined via bidirectional local tblastn and web tblastx searches (Altschul *et al.*, 1990) against NCBI’s non-redundant protein sequences and nucleotide databases (NCBI Resource Coordinators, 2016). Trimmed means of M-values (TMM)-normalized transcripts per million (TPM) values of each sequenced metatranscriptomic sample (R1, R2 and R3) are presented with the averages and standard deviations (SD) across samples. The total average depths of coverage of each transcript for each group of metagenomic samples sequenced on MiSeq, HiSeq, and Nanopore platforms are also presented.

| **Transcript**  **length** | **Closest protein homolog** | **Closest related organism** | **TMM-Normalized TPM** | | | | | **Depth** | | |
| --- | --- | --- | --- | --- | --- | --- | --- | --- | --- | --- |
|  |  |  | **R1** | **R2** | **R3** | **Average** | **SD** | **MiSeq** | **HiSeq** | **Nanopore** |
| 751 | Assimilatory nitrate reductase | *Ca.* T. endoloripes | 0.3 | 0.1 | 0.1 | 0.2 | 0.1 | 0 | 0 | 0 |
| 2484 | NAD(P)H-dependent assimilatory nitrite reductase | *Ca.* T. endoloripes | 0.4 | 0.1 | 0.1 | 0.2 | 0.2 | 0 | 0.1 | 0.3 |
| 753 | FMN-binding glutamate synthase family protein | *Ca.* T. endoloripes | 0.4 | 0.1 | 0.2 | 0.2 | 0.1 | 0 | 0 | 0.03 |
| 4390 | Homocitrate synthase, serine-O-acetyltransferase, NifWZM | *Ca.* T. endoloripes | 1.1 | 0.2 | 0.9 | 0.7 | 0.4 | 0 | 0 | 0.02 |
| 2146 | NifUS | *Ca.* T. endoloripes | 0.5 | 0.2 | 0.3 | 0.3 | 0.2 | 0 | 0 | 0.04 |
| 4373 | NifE | *Ca.* T. endoloripes | 0.3 | 0.1 | 0.3 | 0.2 | 0.1 | 0 | 0.06 | 0.02 |
| 1125 | NifNX | *Ca.* T. endoloripes | 0.4 | 0.1 | 0.2 | 0.2 | 0.1 | 0 | 0 | 0.03 |
| 5028 | NifDKT | *Ca.* T. endoloripes | 0.5 | 0.1 | 0.3 | 0.3 | 0.2 | 0 | 0 | 0.01 |
| 1169 | NifH | *Ca.* T. endoloripes | 0.7 | 0.2 | 0.6 | 0.5 | 0.3 | 0 | 0 | 0.03 |
