# Supplementary material for: Taxonomic and functional heterogeneity of the gill microbiome in a symbiotic coastal mangrove lucinid species
Source: ISME J. 2018 Dec 5;13(4):902–20. doi: 10.1038/s41396-018-0318-3 (PMC6461927; doi:10.1038/s41396-018-0318-3)
Supplement: Supplementary file 14 — Table S5 [file 41396_2018_318_MOESM14_ESM.docx]

**Table S5.** Summary of local tblastn (Altschul *et al.*, 1990) search results querying translated nitrogen fixation genes in sequenced lucinid endosymbionts *Ca.* Thiodiazotropha endoloripes (Petersen *et al.*, 2016) and *Ca.* Thiodiazotropha endolucinida (Konig *et al.*, 2016) against unbinned *P. pectinatus* assemblies. Query sequences were obtained from NCBI’s GenPept sequence database (NCBI Resource Coordinators, 2016). The % identity and expect value (e-value) of the best hit from each query is presented in this table.

| **Query** | **Accession Number** | **Organism** | **# Hits** | **% Identity** | **E-value** | **Alignment Length** |
| --- | --- | --- | --- | --- | --- | --- |
| Nitrogenase cofactor biosynthesis protein NifB | WP_069124667 | *Ca.*  T. endolucinida | 1 | 22.69 | 8 x 10^-5^ | 216 |
| Nitrogenase iron protein | WP_069124654 | *Ca.*  T. endolucinida | 12 | 22.81 | 7 x 10^-7^ | 263 |
| Nitrogen fixation negative regulator NifL | ODB98656 | *Ca.*  T. endoloripes | 221 | 40.52 | 1 x 10^-20^ | 116 |
| Nitrogen fixation negative regulator NifL | ODB99078 | *Ca.*  T. endoloripes | 211 | 40.52 | 3 x 10^-20^ | 116 |
| Nitrogen fixation negative regulator NifL | ODJ87650 | *Ca.*  T. endolucinida | 208 | 38.98 | 5 x 10^-19^ | 118 |
| Nitrogen fixation protein VnfA | ODJ89372 | *Ca.*  T. endolucinida | 176 | 42.11 | 1 x 10^-68^ | 342 |
| Nitrogen fixation protein NifM | ODJ87604 | *Ca.*  T. endolucinida | 24 | 25.77 | 1 x 10^-8^ | 194 |
| Nitrogen fixation protein NifM | ODB97808 | *Ca.*  T. endoloripes | 18 | 24.74 | 1 x 10^-7^ | 194 |
| Nitrogenase iron protein | WP_069005827 | *Ca.*  T. endoloripes | 12 | 22.81 | 5 x 10^-7^ | 263 |
| Nitrogenase iron protein NifH | ODJ87633 | *Ca.*  T. endolucinida | 12 | 22.81 | 7 x 10^-7^ | 263 |
| Nitrogen fixation protein | ODC01960 | *Ca.*  T. endoloripes | 11 | 54.63 | 4 x 10^-30^ | 108 |
| Nitrogen fixation protein | ODB98544 | *Ca.*  T. endoloripes | 11 | 52.78 | 1 x 10^-28^ | 108 |
| Nitrogen fixation protein FixH | ODC01716 | *Ca.*  T. endoloripes | 11 | 53.99 | 4 x 10^-61^ | 163 |
| Nitrogenase cofactor biosynthesis protein NifB | WP_069014752 | *Ca.*  T. endoloripes | 1 | 24.54 | 2 x 10^-5^ | 216 |
| Nitrogenase cofactor biosynthesis protein NifB | WP_069006074 | *Ca.*  T. endoloripes | 1 | 24.54 | 2 x 10^-5^ | 216 |
| Nitrogenase FeMo cofactor synthesis FeS core scaffold and assembly protein NifB | ODJ87648 | *Ca.*  T. endolucinida | 1 | 22.69 | 9 x 10^-5^ | 216 |
| Nitrogen fixation protein NifT | ODJ87630 | *Ca.*  T. endolucinida | 0 | NA | NA | NA |
| Nitrogen fixation protein NifW | ODB97810 | *Ca.*  T. endoloripes | 0 | NA | NA | NA |
| Nitrogen fixation protein NifX | ODB97826 | *Ca.*  T. endoloripes | 0 | NA | NA | NA |
| Nitrogen fixation protein NifZ | ODJ87605 | *Ca.*  T. endolucinida | 0 | NA | NA | NA |
| Nitrogen fixation protein NifZ | ODB98653 | *Ca.*  T. endoloripes | 0 | NA | NA | NA |
| Nitrogen fixation protein NifZ | ODB97809 | *Ca.*  T. endoloripes | 0 | NA | NA | NA |
| Nitrogenase FeMo cofactor synthesis molybdenum delivery protein NifQ | ODJ87641 | *Ca.*  T. endolucinida | 0 | NA | NA | NA |
| Nitrogenase iron-molybdenum cofactor biosynthesis protein NifE | WP_069124645 | *Ca.*  T. endolucinida | 0 | NA | NA | NA |
| Nitrogenase iron-molybdenum cofactor biosynthesis protein NifE | ODJ87623 | *Ca.*  T. endolucinida | 0 | NA | NA | NA |
| Nitrogenase iron-molybdenum cofactor biosynthesis protein NifE | WP_069024648 | *Ca.*  T. endoloripes | 0 | NA | NA | NA |
| Nitrogenaseiron-molybdenum cofactor biosynthesis protein NifN | WP_069124644 | *Ca.*  T. endolucinida | 0 | NA | NA | NA |
| Nitrogenase iron-molybdenum cofactor biosynthesis protein NifN | ODJ87622 | *Ca.*  T. endolucinida | 0 | NA | NA | NA |
| Nitrogenase iron-molybdenum cofactor biosynthesis protein NifN | WP_069014456 | *Ca.*  T. endoloripes | 0 | NA | NA | NA |
| Nitrogenase iron-molybdenum cofactor biosynthesis protein NifX | ODJ87621 | *Ca.*  T. endolucinida | 0 | NA | NA | NA |
| Nitrogenase iron-molybdenum cofactor biosynthesis protein NifY | ODJ87628 | *Ca.*  T. endolucinida | 0 | NA | NA | NA |
| Nitrogenase molybdenum-iron protein alpha chain | WP_069124653 | *Ca.*  T. endolucinida | 0 | NA | NA | NA |
| Nitrogenase molybdenum-iron protein subunit alpha NifD | ODJ87632 | *Ca.*  T. endolucinida | 0 | NA | NA | NA |
| Nitrogenase molybdenum-iron protein subunit beta | WP_069124652 | *Ca.*  T. endolucinida | 0 | NA | NA | NA |
| Nitrogenase molybdenum-iron protein subunit beta | WP_069005826 | *Ca.*  T. endoloripes | 0 | NA | NA | NA |
| Nitrogenase molybdenum-iron protein subunit beta NifK | ODJ87631 | *Ca.*  T. endolucinida | 0 | NA | NA | NA |
| Putative nitrogen fixation protein | ODJ87619 | *Ca.*  T. endolucinida | 0 | NA | NA | NA |
| Putative nitrogen fixation protein | ODJ87618 | *Ca.*  T. endolucinida | 0 | NA | NA | NA |
| Putative nitrogen fixation protein FixT | ODB97838 | *Ca.*  T. endoloripes | 0 | NA | NA | NA |
