# Supplementary material for: Taxonomic and functional heterogeneity of the gill microbiome in a symbiotic coastal mangrove lucinid species
Source: ISME J. 2018 Dec 5;13(4):902–20. doi: 10.1038/s41396-018-0318-3 (PMC6461927; doi:10.1038/s41396-018-0318-3)
Supplement: Supplementary file 15 — Table S6 [file 41396_2018_318_MOESM15_ESM.docx]

**Table S6.** Summary of transcripts involved in amino acid and B vitamin biosynthesis in *Ca.* Sedimenticola endophacoides, the *Kistimonas*-like species and the *Spirochaeta*-like species. Amino acid names are indicated with three-letter codes. Average and standard deviation values of TMM-normalized TPM across metatranscriptomic samples are presented. “No transcript” indicates pathways present in the MAGs but not transcriptomes, while “no gene” indicates pathways not identified in the MAGs.

| **Category** | **Compound** | **TMM-Normalized TPM** | | |
| --- | --- | --- | --- | --- |
|  |  | ***Ca.* Sedimenticola endophacoides** | ***Kistimonas*-like species** | ***Spirochaeta*-like species** |
| **Branched chain amino acids** | Ile, Val, Leu | 0.3 ± 0.7 | No gene/transcript | No transcript |
| **Other hydrophobic amino acids** | Gly | 0.3 ± 0.3 | No transcript | No transcript |
|  | Ala | 0.6 ± 0.9 | 0.06 ± 0.08 | No transcript |
|  | Pro | 0.09 ± 0.1 | 0.2 ± 0.3 | 0.07 ± 0.1 |
|  | Met | 0.2 ± 0.3 | 0.1 ± 0.2 | No gene |
|  | Trp | 0.6 ± 1 | 0.07 ± 0.07 | 0.05 ± 0.08 |
| **Basic amino acids** | Arg | 0.2 ± 0.2 | 0.08 ± 0.09 | No transcript |
|  | Lys | 0.2 ± 0.5 | No transcript | No transcript |
|  | His | 0.3 ± 0.6 | 0.09 ± 2 | No transcript |
| **Acidic/polar amino acids** | Glu, Glt, Asp, Asn | 0.4 ± 0.8 | No transcript | No transcript |
|  | Ser | 0.1 ± 0.2 | 0.1 ± 0.2 | No transcript |
|  | Thr | 0.3 ± 0.5 | 0.09 ± 0.2 | No transcript |
|  | Cys | 0.2 ± 0.3 | 0.2 ± 0.3 | No transcript |
| **Other amino acids** | Phe, Tyr | 0.09 ± 0.1 | 0.07 ± 0.01 | No transcript |
| **B vitamins** | Vitamin B1 | 0.2 ± 0.3 | No transcript | No transcript |
|  | Vitamin B2 | 0.2 ± 0.3 | 0.07 ± 0.1 | No transcript |
|  | Vitamin B3 | No gene | No gene | No gene |
|  | Vitamin B5 | No gene | No gene | No gene |
|  | Vitamin B6 | 0.2 ± 0.3 | 0.1 ± 0.2 | No transcript |
|  | Vitamin B7 | 0.1 ± 0.2 | 0.08 ± 0.09 | No transcript |
|  | Vitamin B9 | 8 ± 25 | 0.09 ± 0.1 | 0.08 ± 0.1 |
|  | Vitamin B12 | No gene | No gene | No transcript |
