# Supplementary material for: Taxonomic and functional heterogeneity of the gill microbiome in a symbiotic coastal mangrove lucinid species
Source: ISME J. 2018 Dec 5;13(4):902–20. doi: 10.1038/s41396-018-0318-3 (PMC6461927; doi:10.1038/s41396-018-0318-3)
Supplement: Supplementary file 16 — Supplementary Information [file 41396_2018_318_MOESM16_ESM.docx]

# Supplemental Information – Lim *et al.*

# Supplemental Materials and Methods

# Site characterization and porewater geochemistry

After lucinid density at Wildcat Cove was established to be more than one *Phacoides* *pectinatus* specimen per shovel-full of sediment, which was within 3 m of the mangrove-lined shoreline, a sampling area was sectioned off into quadrats, ranging from 0.5-m^2^ to 1-m^2^ over, in general, a 100-m^2^ area. During the 2014 sampling, sediment porewater was obtained from six quadrats (Doty, 2015) by low-flow fluid sampling using stainless steel piezometers installed near to where specimens were recovered, based on previously descried methods (Green-García and Engel, 2012). Standard electrode methods were used to measure dissolved oxygen content from the porewater, as well as pH, temperature, conductivity, and to collect water samples for dissolved ion and total organic carbon concentrations (Doty, 2015). Dissolved sulfide concentrations were obtained colorimetrically using CHEMetrics (Calverton, VA, USA) chemistry and a field spectrophotometer (Green-García and Engel, 2012; Doty, 2015).

# Microscopy and fluorescence in situ hybridization (FISH)

Following paraformaldehyde fixation, gill tissues were washed 3x for five minutes each in artificial sea water (ASW) and stored in the same medium overnight before infiltration with 10% and 25% sucrose in ASW. Tissues were stored at 4°C prior to cryosectioning. Hematoxylin-eosin (H&E) staining and FISH were performed on 5 μm cryosections from tissues cut with Thermo Fisher Scientific’s (Pittsburgh, PA, USA) Microm™ HM550 Cryostat on VistaVision™ HistoBond® Adhesive Slides (VWR, Radnor, PA, USA). Prior to FISH, cryosections were soaked in diethyl pyrocarbonate (DEPC)-treated phosphate-buffered saline (PBS) solution for five minutes and air dried for an hour. Hybridization, washing, counter-staining, and mounting steps were performed in accordance to the Standard FISH protocol (https://www.arb-silva.de/fish-probes/fish-protocols/) available on Silva’s web server (Quast *et al.*, 2013), except that 4',6-diamidino-2-phenylindole (DAPI) counter-staining was extended to 12 minutes, followed by three washes at five minutes each with DEPC-treated PBS solution.

A probe named SED642 (5’-ACCATACTCTAGCCTGCCAG-3’) was designed to hybridize to the *P. pectinatus* endosymbiont, *Ca*. Sedimenticola endophacoides, based on the alignment of full-length 16S rRNA gene sequences extracted from the species’ MAGs with the BangT-642 probe used for the *Bathymodiolus* sp. mussel symbiont (5′-CCTATACTCTAGCTTGCCAG-3′) (Duperron *et al.*, 2005) in ClustalW (Thompson *et al.*, 1994) implemented in BioEdit 7.2.5 (Hall, 1999). The specificity of probe SED642 was evaluated using the NCBI’s Basic Local Alignment Search Tool (BLAST) web tool (Altschul *et al.*, 1990) against the 16S ribosomal RNA sequence database (NCBI Resource Coordinators, 2016). Probe SED642 shared 100% sequence identity with 16S rRNA gene sequences of strains from the genera *Salinispirillum*, *Methylophaga*, *Marinomonas*, *Methylosphaera*, and *Pseudomonas*, and one or more mismatches to a range of other strains predominately within the class Gammaproteobacteria, but also to strains from the Actinobacteria and Firmicutes (**Table S1**). As genera matching probe SED642 have not been previously associated with symbiotic associations in bivalves, the likelihood of false positive signals from these organisms on the *P. pectinatus* gill samples was considered low. Probe SED642 was labelled with Cy5 (Integrated DNA Technologies, Skokie, IL, USA) at the 5’ end. Probes EUB338 (5’-GCTGCCTCCCGTAGGAGT-3’) (Amann *et al.*, 1990) for the general detection of bacteria and its reverse complement NON338 (5’-ACTCCTACGGGAGGCAGC-3’) (Wallner *et al.*, 1993) were both labelled with Cy3 (Integrated DNA Technologies, Skokie, IL, USA) at the 5’ end and used as positive and negative controls, respectively. A range of formamide concentrations between 10% to 35% were tested and signals for all probes were found to be optimal at 30% formamide concentration. Hybridized sections were imaged with Leica’s SPE confocal microscope at the Clemson Light Imaging Facility (Clemson, SC, USA) and the Leica Application Suite (LAS) X software (Buffalo Grove, IL, USA). H&E stained sections were visualized using Leica’s DM750 microscope attached to a Leica ICC50HD camera and the LAS EZ V2.1.0 software (Buffalo Grove, IL, USA).

# 16S rRNA gene library preparation

cDNA was synthesized from DNase-treated (Ambion® Turbo DNA-free™ DNase kit, Life Technologies) RNA with the High-Capacity cDNA Reverse Transcription Kit (Applied iosystems, Foster City, CA, USA). Successful DNase treatment and cDNA synthesis was confirmed with PCR amplification of the V9 region of bacterial 16S rRNA genes (see next section). DNA and cDNA from each sample was diluted to 1 ng/µL with nuclease-free water and amplified with previously developed dual indexes (Kozich *et al.*, 2013). Amplicons were normalized with the SequalPrep™ Normalization Plate Kit, 96-well (Invitrogen, Carlsbad, CA, USA), quantitated with the Qubit™ dsDNA HS assay (Life Technologies, Austin, TX, USA) and pooled for sequencing.

# PCR amplification of the V9 region of bacterial 16S rRNA genes

To check for successful DNase treatment, the V9 region of the 16S rRNA gene in DNAse-treated (Ambion® Turbo DNA-free™ DNase kit, Life Technologies) RNA samples was amplified with previously described universal bacterial primers, 1369F and 1492R (Suzuki *et al.*, 2000) (**Table S2**). PCR reactions were performed in a 10 μl volume consisting of 0.25 µM of each primer and 1x BIO-X-ACT™ Short Mix (Bioline, Taunton, MA, USA). PCR amplification was carried out in the C1000 Touch™ Thermal Cycler (Bio-Rad Laboratories, Hercules, CA, USA) under the following conditions: Initial denaturation at 95°C for 3 minutes, 29 cycles of denaturation at 95°C for 15 seconds, annealing at 53°C for 30 seconds, extension at 72°C for 30 seconds, followed by elongation at 72°C for 5 minutes and cooling at 12°C. Amplicons were run on a 1% (wt/vol) agarose gel, which was stained with ethidium bromide, de-stained with deionized water and visualized under UV with a FOTO/Analyst® FX System (Fotodyne Inc., Hartland, WI, USA).

# Checking for DNA contamination in metatranscriptomic libraries

To check for DNA contamination in sequenced metatranscriptomic libraries, trimmed reads were mapped to a representative, high-quality *Ca.* Sedimenticola endophacoides MAG (N3+P5) with high completeness value and low contamination and strain heterogeneity values (**Table 1**) using Bowtie2 v2.2.7’s (Langmead and Salzberg, 2012) no-mixed, no-discordant, end-to-end, -k 200 and -gbar 1000 options. The read-to-MAG mapping was inspected in SeqMonk v1.42.0 (Babraham Bioinformatics, 2007) for consistent alignments across gene boundaries and directionality bias indicative of DNA contamination. No DNA contamination was observed in the metatranscriptomic libraries.

# Sequencing service providers

16S rRNA gene libraries from the 2014 collection were sequenced by the Duke Center for Genomic and Computational Biology (Durham, NC, USA), while those from the 2017 collection were sequenced at Clemson University, SC, USA. Molecular Research LP (Shallowater, TX, USA) prepared the 2011 and 2013 metagenomic libraries and sequenced them on the MiSeq platforms. Library insert size determination with the Agilent 2100 Bioanalyzer (Agilent Technologies, Santa Clara, CA, USA) and outsourcing of Illumina HiSeq 2500 sequencing were performed by Clemson University Genomics Institute (CUGI; Clemson, SC, USA). Metatranscriptomic libraries were pooled and sequenced by the Duke Center for Genomic and Computational Biology.

# 16S rRNA gene analysis pipeline

All reads were quality trimmed at Q=25 with Mothur v1.39.5 (Schloss *et al.*, 2009) using the trim_seq and remove_seq commands. Reads sequenced at Duke University were additionally matched to the read 1 (GCCGCGGTAA) and read 2 (GGGTNTCTAAT) primers for the V4 region of the 16S rRNA gene to exclude non-target reads and to trim off primer sequences with Mothur’s pcr.seqs command. Surviving reads were processed in Mothur per the software’s MiSeq SOP (Schloss *et al.*, 2009). The alignment step was modified to include the reverse complement of each sequence and report the better alignment (flip=T). Processed sequences were clustered into Operational Taxonomic Units (OTUs) and these were classified taxonomically against Silva v132 (Quast *et al.*, 2013) at 80% and 0% bootstrap confidence. Because the sequencing depths per sample were uneven, and ranged from 926 sequences to 27,323 sequences, the data was normalized by sub-sampling to 1,269 sequences (the smallest four-digit number in the dataset, eliminating two DNA samples from the 2014 collection. Using Mothur (Schloss *et al.*, 2009), the relative abundance of each OTU in each sample was computed by scaling its total abundance with the total number of sequences in the sample, and the Good’s coverage estimator (Good, 1953) for each sample was calculated using the formula: C = 1 – (number of OTUs sampled once / total number of individuals). 16S rRNA gene sequences used for phylogenetic analysis were retrieved from literature (Durand and Gros, 1996; Duperron *et al.*, 2007; Green-García, 2008; Brissac *et al.*, 2011) and from the GenBank database (Benson *et al.*, 2014) via keyword searches. All sequences were aligned with ClustalW (Thompson *et al.*, 1994) implemented in BioEdit 7.2.5 (Hall, 1999), and positions with gaps were manually trimmed off. MEGA6 (Tamura *et al.*, 2013) was used to predict the best nucleotide substitution model. The Kimura 2-parameter model (Kimura, 1980) with discrete Gamma distribution modeling of the evolutionary rate differences among sites (5 categories, with +G, parameter = 0.3382) was used to generate a maximum likelihood tree with 1,000 bootstrap replicates.

# qPCR procedures to assess gill microbiome diversity

Universal primers 27F (Lane, 1991) and 1391R (Turner *et al.*, 1999) targeting the 16S rRNA gene were used to amplify total DNA extracted from one *P. pectinatus* gill sample. The amplified gene fragment was ligated into the pGEM®-T Vector System (Promega, Madison, WI, USA), followed by transformation by JM109 High-Efficiency Competent Cells (Promega) on lysogeny broth (LB)/ampicillin plates incubated at 37°C for 24 hours. White colonies were amplified with universal M13 forward and reverse primers targeting binding sites within the vector. PCR reactions were performed as previously described in this document under the following conditions: Initial denaturation at 95°C for 5 minutes, 29 cycles of denaturation at 95°C for 30 seconds, annealing at 55°C for 30 seconds, extension at 72°C for 30 seconds, followed by elongation at 72°C for 5 minutes and cooling at 12°C. Amplicons were run and imaged on a 1% (wt/vol) agarose gel as previously described in this document. Three transformed colonies were re-grown on another LB/ampicillin plate, re-tested with PCR and grown on liquid LB/ampicillin broth for 24 hours at 37°C. Plasmids were extracted from liquid broth using the QIAprep Spin Miniprep Kit (Qiagen, Valencia, CA, USA), linearized with FastDigest® *Nco*I or *Nde*I (Thermo Fisher Scientific, Waltham, MA, USA) and quantified with Qubit™ dsDNA assays (Life Technologies, Austin, TX, USA). PCR-amplified gene fragments of the linearized plasmids were sequenced using the Sanger method by CUGI (Clemson, SC, USA). Sequenced inserts compared against OTU sequences identified by 16S rRNA gene sequencing showed perfect match to the *Sedimenticola*-like OTU 1 sequence. Linearized plasmids were serially diluted and used to generate standard curves for absolute qPCR. qPCR primers for the 16S rRNA gene targeting the *Sedimenticola*-like OTU 1 (1417F and 1580R; **Table S2**) were designed using the Primer3 software (Untergasser *et al.*, 2012) integrated in Geneious v8.0 (Kearse *et al.*, 2012) based on the Sanger-sequenced insert sequences. Cloning and qPCR primers for 16S rRNA genes targeting the *Kistimonas*-like OTU2 (90F and 231R; positions based on V4 region) and *Spirochaeta*-like OTU5 (15F and 226R; positions based on V4 region; **Table S2**) were designed based on their OTU sequences using the same software. For each qPCR reaction, all samples and standards were amplified in triplicate with a 10 μl volume consisting of 0.25 µM of each primer and 1x SsoAdvanced™ Universal SYBR® Green Supermix (Bio-Rad Laboratories). All qPCR amplifications were carried out in the C1000 Touch™ Thermal Cycler (Bio-Rad Laboratories) under the following conditions: Initial denaturation at 95°C for 3 minutes, 29 cycles of denaturation (33 cycles for *Spirochaeta*-like OTU5 amplification) at 95°C for 15 seconds, annealing (**Table S2**) for 30 seconds, extension at 72°C for 30 seconds, followed by elongation at 72°C for 5 minutes. Data analysis was performed with the CFX Manager software (Bio-Rad Laboratories) and all copy numbers were normalized to the amount (ng) of input DNA.

# Sequence and genome quality assessment

Adapter removal and quality trimming (Q=30) of all Illumina-sequenced reads were performed using Cutadapt v1.11 (Martin, 2011), followed by Sickle v1.33 (Joshie and Fass, 2011). Read qualities pre- and post-trimming were assessed with FastQC v0.11.5 (Babraham Bioinformatics, 2010). The quality of each metagenome-assembled genome (MAG; size, number of contigs, GC content, and Nx values) was evaluated with the Quality Assessment Tool for Genome Assemblies (QUAST, v4.5) (Gurevich *et al.*, 2013). Genome lineage, completeness, contamination, and strain heterogeneity statistics were estimated by CheckM v1.0.9 (Parks *et al.*, 2015), based on a set of lineage-specific single-copy marker genes. Using reference datasets specific to each lineage identified by CheckM (Parks *et al.*, 2015), the completeness of each MAG was further evaluated with Benchmarking Universal Single-Copy Orthologs (BUSCO) v3.0.1 (Simao *et al.*, 2015). Based on the completeness, contamination, presence of the 23S, 16S, and 5S rRNA genes and the number of tRNAs in each MAG, a quality measure was assigned according to the Minimum Information about a Metagenome-Assembled Genome (MIMAG) standard (Bowers *et al.*, 2017). Host (eukaryotic) genomic contamination was further assessed with MEtaGenome ANalyzer (MEGAN) community edition v6.6.4 (Huson *et al.*, 2007), which performs taxonomic assignment from web blastn (Altschul *et al.*, 1990) search results of contig sequences in each draft assembly. Ensemble binning by MetaBat (Kang *et al.*, 2015) largely removed contaminating reads from the host genome, except for the spirochete MAG where eight eukaryotic contigs were detected out of 185 contig sequences. These contigs were removed manually from the spirochete MAG with no impact on the genome quality. Ensemble binning also failed to bin the gammaproteobacterial 16S rRNA gene sequences from the Illumina assemblies. These sequences were recovered from initial, unbinned assemblies by local blastn (Altschul *et al.*, 1990) searches with the NCBI BLAST 2.2.30+ package (NCBI Resource Coordinators, 2016) using 16S rRNA gene sequences extracted from the Nanopore co-assemblies as query. Matched contigs containing the 16S rRNA sequences were evaluated with web blastn (Altschul *et al.*, 1990) searches and added to their corresponding MAGs with no impact on the quality of these MAGs.

# Sequencing depth analyses

MAG depth profiles were generated by mapping trimmed reads from each sample back to the representative, most complete *Ca.* Sedimenticola endophacoides (N1 + N3 + P5), *Kistimonas*-like sp. (P2) and *Spirochaeta*-like sp. (P3) MAGs using Bowtie2 v2.2.7 (very sensitive local and dovetail mode) (Langmead and Salzberg, 2012) and SAMtools v0.1.19 (Li *et al.*, 2009). The depth of coverage of each contig in a MAG was summarized with MetaBat’s v0.32.4 (Kang *et al.*, 2015) jgi_summarize_bam_contig_depths script, normalized by dividing each value to the expected genome size extrapolated from CheckM (Parks *et al.*, 2015) and BUSCO (Simao *et al.*, 2015) predictions and averaged for the entire MAG.

# Sequence comparisons with published reference genomes

Reciprocal average nucleotide identity (ANI) and average amino acid identity (AAI) values estimating genetic relatedness between strains and species (Konstantinidis and Tiedje, 2005) were calculated using

DOE Joint Genome Institute’s whole-genome based average nucleotide identity (gANI) tool (Varghese *et al.*, 2015) and CompareM v0.0.23 (Roux *et al.*, 2015), respectively. Calculated ANI and AAI values were averaged and visualized with the heatmaply package in R (https://cran.r-project.org/web/packages/heatmaply/index.html) (R Core Team, 2016). Reference genomic/MAG sequences used for phylogenomic analysis were retrieved from NCBI’s GenBank (Benson *et al.*, 2014) and Genome databases (NCBI Resource Coordinators, 2016) via keyword searches. Phylogenomic analysis was performed with scripts within phylogenomics-tools (Seah, 2014), which uses AMPHORA2 (Wu and Eisen, 2008) to extract marker genes conserved in bacteria. Ten single-copy genes (*dnaG*, *frr*, *nusA*, *pgk*, *pyrG*, *rplM*, *rplS*, *rpmA*, *rpsB*, *rpsI*) present in all compared genomes/MAGs were aligned with Muscle v3.8.31 (Edgar, 2004). No marker gene was detected in the foot assembly. Each gene alignment was visually inspected to remove poorly aligned regions. The concat_align.pl script then concatenates all alignments into a partitioned alignment and predicts the best protein substitution model for each marker gene (LGF for *dnaG*, *nusA*, *pgk*; LG for *frr*, *pyrG*, *rplM*, *rplS*, *rpsB,* and *rpsI;* WAG for *rpmA*). Maximum likelihood trees with aLRT (approximate likelihood-ratio test) SH-like support values (Anisimova and Gascuel, 2006) were inferred for each partition and the combined partitions with RAxML v7.7.2 (Stamatakis, 2014). Reference protein sequences for phylogenetic classification of sulfide:quinone oxidoreductase (Sqr) and related flavocytochrome sulfide dehydrogenase (FCC) proteins were obtained from (Marcia *et al.*, 2010) and NCBI’s nr (non-redundant) database (NCBI Resource Coordinators, 2016), then aligned using ClustalW (Thompson *et al.*, 1994) implemented in BioEdit 7.2.5 (Hall, 1999). MEGA v6.06 (Tamura *et al.*, 2013) was used to predict the best protein substitution model for the alignment, the LG model (Le and Gascuel, 2008), with discrete Gamma distribution modeling of the evolutionary rate differences among sites (5 categories, with +G, parameter = 5.43). Based on the model, an unrooted maximum likelihood tree with 100 bootstrap replicates was generated. Positions with less than 95% site coverage were deleted. Classification of ribulose-1,5-bisphosphate carboxylase/oxygenase (RuBisCO) protein and hydrogenase sequences were performed via the NCBI’s Basic Local Alignment Search Tool (BLAST) web tool (Altschul *et al.*, 1990) against annotated RuBisCO sequences in the nr database (NCBI Resource Coordinators, 2016) and hydrogenase sequences in the deep-sea scaly-foot snail esophageal gland symbiont (Nakagawa *et al.*, 2014). Hydrogenase class definitions in Peters *et al.* (2015) were used for classification.

# PCR procedures to validate metagenomics and metatranscriptomics results

To validate nitrogen assimilation functions in the *Kistimonas*-like species, PCR primers targeting the assimilatory nitrate reductase (*nas*; 229F and 364R) and assimilatory nitrite reductase (*nit*; 454F and 580R) genes were designed (**Table S2**). To validate vitamin B12 synthesis functions in the *Spirochaeta*-like species, PCR primers targeting the cobyrinate a,c-diamide synthase (*cbiA*; 298F and 394R) gene previously used to detect potential vitamin B12 biosynthesizers in the Ross Sea (Bertrand *et al.*, 2011) and the cobalt-precorrin-5A hydrolase (*cbiG*; 272F and 410R) genes were designed (**Table S2**). PCR primers for the *btuF* gene (692F and 794R) encoding vitamin B12-binding protein involved in transport in the *Spirochaeta*-like species were also designed (**Table S2**). PCR reactions were performed as previously described in this document under the following conditions: Initial denaturation at 95°C for 2 minutes, 33 cycles of denaturation at 95°C for 15 seconds, annealing (**Table S2**) for 30 seconds, extension at 72°C for 30 seconds, followed by elongation at 72°C for 5 minutes and cooling at 12°C. Amplicons were run and imaged on a 1% (wt/vol) agarose gel as previously described in this document.

# Supplemental Results

# Foot microbiome diversity in *P. pectinatus*

*Phacoides pectinatus* foot DNA samples were dominated by one *Christensenella*-like OTU (OTU3) belonging to the class Clostridia at average 51 ± 21% relative abundance (**Figure 1a**). Low occurrences of the *Sedimenticola*-like OTU1 (average 25 ± 9% relative abundance), the *Kistimonas*-like OTU2 (average 8 ± 6% relative abundance) and the *Spirochaeta*-like OTU5 (average 0.5±0.3% relative abundance) were also detected in all foot specimens (**Figure 1a**). No bacterial phylogenetic marker gene or 16S rRNA gene sequence was detected in the foot metagenome. 0.02% of foot metagenomic reads mapped to the *Ca.* Sedimenticola endophacoides MAG, 0.001% mapped to the *Kistimonas*-like MAG and 2% mapped to the *Spirochaeta*-like MAG.

# Composition of unbinned metagenomic contigs

Out of 527,385 total unbinned contigs from the deeply sequenced gill metagenome, gene/protein homologs were only predicted in 25,670 contigs (5%). ~94% of the homologs were eukaryotic, ~4% were bacterial, ~1% were viral and~ 0.2% were archaeal. ~75% of the bacterial homologs belonged to the phylum Proteobacteria, while only ~2% classified to the phylum Spirochaetes. Of all proteobacterial homologs, ~70% belonged to the class Gammaproteobacteria.

# Composition of gill metatranscriptomes

57% of the 1,329,218 unclassifiable transcripts mapped to the unbinned contigs in the deeply sequenced metagenomic sample and only <0.1% of these mapped to the MAGs generated in this study. Among the 91,465 transcript clusters (loosely equivalent to genes), ~51% were eukaryote-related, ~45% were bacteria-related, ~2% were archaea-related, and ~2% were virus-related. About 2.87% of all assembled transcripts were predicted to be rRNA.

# Highly expressed bacteria-related housekeeping and stress response transcript clusters

Based on Uniprot’s annotations (The UniProt Consortium, 2015), many of the top 30 most abundant bacteria-related transcript clusters were involved in housekeeping functions, including DNA repair (exodeoxyribonuclease V subunit gamma RecC), transcriptional regulation (sigma-54-dependent Fis family transcriptional regulator), protein folding (filamentous temperature-sensitive ATP-dependent zinc metalloprotease FtsH and its modulator HflK), signaling (diguanylate cyclase response regulator and two-component response regulator; diguanylate cyclase), folate-dependent one-carbon metabolism or biosynthesis (5-formyltetrahydrofolate cyclo-ligase), cofactor synthesis (ubiquinone/menaquinone biosynthesis C-methyltransferase UbiE), and stress response (heat shock proteins, extracytoplasmic function RNA polymerase sigma-E factor RpoE, molecular chaperones ClpB and DnaK; **Figure 3a**).

# Nitrogen fixation and assimilatory nitrate and nitrite reduction in *Ca.* Sedimenticola endophacoides

Though transcripts homologous to most nitrogen fixation proteins and assimilatory nitrate and nitrite reductases in *Ca.* Thiodiazotropha spp. were identified in the gill metatranscriptomes, normalized metagenomic read coverages of these transcripts were low, averaging 0 ± 0 for MiSeq sequenced reads, 0.02 ± 0.01 for Nanopore sequenced reads and 0.02 ± 0.04 for HiSeq sequenced reads (**Table S4**). In comparison, metagenomic read depths of other nitrogen metabolism transcripts averaged at 80 ± 381 for MiSeq sequenced reads, 0.009 ± 0.01 for Nanopore sequenced reads and 25,659 ± 143,903 for HiSeq sequenced reads.

# Secretion systems in *Ca.* Sedimenticola endophacoides

Although *lapBCE* genes involved in the type I secretion of the adhesin LapA were identified in MAGs, only the *lapC* transcript cluster was detected at a low average TPM of 0.2 ± 0.3 TPM. Transcript clusters encoding the general secretory pathway protein A (GspA; average 12 ± 15 TPM) and the pullulanase secretion protein E (average 0.1 ± 0.2 TPM), both of which are part of the type II secretion system, were identified. Gene homologs of type II secretion GspABCDFGHIJKLMN proteins in *Ca.* Thiodiazotropha endoloripes could only be mapped to the *P. pectinatus* gill metatranscriptomes, but not the metagenomes. These transcripts had very low metagenomic read depths, averaging 0.0004 ± 0.001 for MiSeq sequenced reads, 0.03 ± 0.01 for Nanopore sequenced reads, and 0.05 ± 0.08 for HiSeq sequenced reads. The *tadAB* and *tadD* genes, which could be part of the type IV pilus and the type II secretion system (Tomich *et al.*, 2007), were expressed in *Ca.* Sedimenticola endophacoides at average TPM of 0.3 ± 0.1, 0.06 ± 0.06, and 0.2 ± 0.2, respectively. Transcript clusters for TatABC (average 0.4 ± 0.6 TPM) and SecYEG (average 0.2 ± 0.2 TPM) proteins within the twin-arginine translocation, and secretory protein export systems that may be linked to the type II secretion system (Nivaskumar and Francetic, 2014), were also observed in *Ca.* Sedimenticola endophacoides. Genes encoding the export apparatus protein and inner and outer membrane proteins of the type III secretion system were detected in *Ca.* Thiodiazotropha endoloripes. Protein homologs of these genes were mapped to a single 23,025 bp transcript within the *P. pectinatus* metatranscriptomes with only 0, 0.03 and 0.03 and MiSeq, Nanopore, and HiSeq metagenomic read depths. Nevertheless, transcripts encoding flagellar export proteins homologous to components of the type III secretions system (Diepold and Armitage, 2015) were identified in *Ca.* Sedimenticola endophacoides. Type VI secretion proteins annotated in *Ca.* Thiodiazotropha spp. MAGs, but not in MAGs of *Ca.* Sedimenticola endophacoides, were mapped to *P. pectinatus* metatranscriptomes with higher metagenomic read depths of 0.8 ± 3 for MiSeq sequenced reads, 0.03 ± 0.01 for Nanopore sequenced reads, and 296 ± 1,006 for HiSeq sequenced reads.

# Other genetic functions in Ca. Sedimenticola endophacoides

Motility-related genes involving the type VI pilus (average 10 ± 37 TPM), flagella (average 2 ± 4 TPM), and chemotaxis proteins (average 2 ± 2 TPM) were observed in transcriptomes of *Ca.* Sedimenticola endophacoides. Phosphate uptake was regulated via proteins encoded by the phosphate regulon (*pho*; average 0.8 ± 1 TPM) and phosphate transporter operon (*pst*; average 0.6 ± 0.9 TPM). The species could also potentially synthesize and hydrolyze inorganic polyphosphate through the activity of polyphosphate kinase and exopolyphosphatase (average 0.9 ± 1 TPM) and/or other phosphatases (average 2 ± 3 TPM). For iron scavenging, *Ca.* Sedimenticola endophacoides, like *Ca.* Thiodiazotropha spp., encoded and expressed the ferrous iron transport protein B (FeoB; average 1 ± 1 TPM), the TonB-ExbB-ExbD ferric siderophore transport system (average 0.06 ± 0.09 TPM), and the ferric uptake regulation protein FUR (average 0.2 ± 0.3 TPM). Transcript clusters for the biosynthesis of all 20 essential amino acids (**Table S6**), most B vitamins (except vitamins B3, B5 and B12; **Table S6**), coenzyme A (average 0.4 ± 0.7 TPM), tetrapyrroles (heme and siroheme; average 0.9 ± 4 TPM) and NAD and NADP cofactors (average 4 ± 9 TPM) were also identified in the species’ transcriptomes.

# Other genetic functions in *Kistimonas*-like species

Eight gill cDNA samples were amplified for the presence of assimilatory nitrate reductase (*nas*) and the assimilatory nitrite reductase (*nit*) genes. Although *nas* showed no amplification, *nit* showed amplification in all samples tested. Transcriptomes of the *Kistimonas*-like species included transcript clusters involved in the transport of metal ions (sodium, potassium and cadmium; average 0.2 ± 0.3 TPM), long-chain fatty acid (average 0.2 ± 0.3 TPM), drugs (average 0.08 ± 0.1 TPM), serine (average 0.08 ± 0.1 TPM), leucine (average 0.07 ± 0.2 TPM), and other substrates (average 0.1 ± 0.1 TPM). Transcript clusters encoding the outer membrane protein OmpW (average 0.5 ± 0.4 TPM), TolC (average 0.3 ± 0.08 TPM), a type I secretion outer membrane protein (average 0.02 ± 0.04 TPM), and Omp assembly factors (average 0.1 ± 0.1 TPM) were also expressed in this species. Other transport-related functions identified in the species’ MAG, but not transcriptomes, included substrates such as urea cycle products spermidine and putrescine and hemin. Bacteriocin processing genes were also detected in the species’ MAG, but not transcriptomes. Like *Ca.* Sedimenticola endophacoides, the *Kistimonas*-like species expressed genes for the biosynthesis of vitamin B2, B6, B7 and B9 (**Table S6**). Vitamin B1 synthesis genes were identified in the species’ MAGs, but not transcriptomes, while genes for the biosynthesis of vitamins B3, B5, and B12 were not sequenced in the MAGs. Transcript clusters for the biosynthesis of proline (gamma-glutamyl phosphate reductase), cysteine, methionine, threonine and homoserine (homoserine dehydrogenase), serine (phosphoserine aminotransferase), histidine (phosphoribosyl-AMP cyclohydrolase), arginine, tryptophan, and alanine were detected in the bacterial species’ transcriptomes (**Table S6**). On the other hand, biosynthetic genes for glycine, glutamine, glutamate, asparagine, aspartate, lysine, and leucine were identified in the MAGs, but not in the transcriptomes (**Table S6**). In the *Kistimonas*-like MAG, genes for *de novo* biosynthesis of the branched chain amino acids isoleucine and valine were not sequenced.

# Other genetic functions in *Spirochaeta*-like species

Transcript clusters for many amino acid biosynthetic pathways in the *Spirochaeta*-like species were not detected in its transcriptomes (**Table S6**), although these were predicted in its MAG. The methionine biosynthesis pathway was not sequenced in the *Spirochaeta*-like species MAG, but methionine degradation genes were expressed in its transcriptome (average 0.2 ± 0.2 TPM; **Table S6**). Genes for vitamins B1, B2, B6, B7, B9, and B12 biosynthesis were annotated in the species MAG, but only transcript clusters for vitamin B9 synthesis were observed (average 0.08 ± 0.1; **Table S6**). Genes for the transmembrane and substrate-binding component of a vitamin B1 ABC transporter, and vitamin B7 uptake proteins BioM and BioY were detected in the *Spirochaeta*-like species MAG, but not transcriptomes. The *Spirochaeta*-like MAG encoded for a nearly complete pathway (missing *cbiJ* and *cbiET*) for anaerobic vitamin B12 biosynthesis (Moore and Warren, 2012) not annotated in *Ca.* Sedimenticola endophacoides and the *Kistimonas*-like species. Although their corresponding transcripts were not detected in the species’ transcriptomes, PCR targeting the cobyrinate a,c-diamide synthase (*cbiA*) and cobalt-precorrin-5A hydrolase (*cbiG*) transcripts showed amplification in 18 out of 19 gill cDNA samples and 7 out of 8 gill cDNA samples tested, respectively. We also observed PCR amplification of the *btuF* gene encoding a vitamin B12-binding protein involved in transport in eight gill cDNA samples tested. Genes for a possible nitrogen assimilation pathway with NADH-dependent glutamate synthase and purine salvage (incomplete pathway) through xanthine uptake and metabolism (Xi *et al.*, 2000) were also identified in the species’ MAGs, but not transcriptomes. Respiration-related genes were not identified in both the species’ MAG and transcriptomes.

# Supplemental Discussion

# Host selection via bactericidal compounds

Host-related transcript clusters for the synthesis of bactericidal compounds encoding H_2_O_2_-generating flavoenzymes (Ehara *et al.*, 2002; Guo *et al.*, 2012), aplysianin-A (Takamatsu *et al.*, 1995), and nitric oxide (Davidson *et al.*, 2004) were weakly to moderately expressed in *P. pectinatus*. Oxidative stress-mediated symbiont selection involving nitric oxide (Davidson *et al.*, 2004) and antibacterial hypohalous acid generated from H_2_O_2_ and halide ions have been reported in the *Eupyrmna*-*Vibrio* symbiosis (Weis *et al.*, 1996; Small and McFall-Ngai, 1999; Schleicher and Nyholm, 2011). Gill microbiome defense to oxidative stressors likely involves weakly-expressed transcripts that detoxify hydrogen peroxide, free radicals, and nitric oxide in *Ca.* Sedimenticola endophacoides and strongly-expressed transcripts encoding heat shock proteins in *Ca.* Sedimenticola endophacoides, and the *Kistimonas*-like and *Spirochaeta*-like species. High abundances of heat shock proteins and chaperones were also observed in the *B. azoricus* symbionts and hypothesized to be an indication of the thioautotrophic symbiont’s transition into an obligate symbiont (Ponnudurai *et al.*, 2017), although other studies have described the protective functions of heat shock proteins against oxidative stress (Kalmar and Greensmith, 2009).

# Supplemental Figure Legends

**Figure S1.** Map showing location of the sampling site, with a close-up view of Wildcat Cove, Florida, USA (Insert A).

**Figure S2**. (A) Heatmap of two-way, pairwise average amino acid identities (AAI) comparisons and (B) phylogenomic tree of MAGs sequenced in this study (red) in relation to published thioautotrophic symbionts of lucinid clams (blue) and other symbiotic and free-living bacteria. The outgroup used in (B) was *Desulfurobacterium thermolithotrophum* from phylum Aquificae and the scale bar indicates 0.2 substitution per site.

**Figure S3.** Fluorescence in situ hybridization (FISH) images of a *P. pectinatus* gill transverse section showing (A) bacteriocytes hybridized with probe SED642 specific for *Ca.* Sedimenticola endophacoides (red), (B) bacteriocytes hybridized with universal probe EUB338 (Amann et al., 1990) for bacterial species (green), (C) bacteriocytes stained with DAPI (blue), (D) differential interference contrast view, (E) overlay view, (F) a light micrograph of another gill section stained with hematoxylin and eosin as a reference for tissue structural integrity and morphology.

**Figure S4.** Bootstrap consensus tree of the ten most abundant 16S rRNA gene OTUs identified in this study (red text), in relation to lucinid (blue text), bivalve, tubeworm, and termite symbionts and free-living bacteria. GenBank (Benson et al., 2014) accession numbers are indicated in square brackets and bootstrap values >70% are shown. The outgroup used was *Desulfurobacterium thermolithotrophum* from phylum Aquificae.

**Figure S5**. Plots showing the sum of fragments mapped to the (A) metatranscriptome assembled *de novo* by Trinity (Haas *et al.*, 2013) for each sequenced sample (R1, R2 and R3) and (B) pairwise Pearson correlations between each sequenced sample. For both plots, the count matrix was transformed to counts per million, followed by a log_2_ transformation.

**Figure S6.** Log_2_-transformed TMM-normalized TPM of gene products of the 30 most abundantly expressed transcript clusters for each sequenced metatranscriptomic sample (R1, R2 and R3) in whole *Phacoides pectinatus* gill metatranscriptomes. Abbreviations: UDP, uridine diphosphate.

**Figure S7**. Top 30 most represented gene ontology (GO) terms (Harris *et al.*, 2004) in the (A) cellular component, (B) biological processes and (C) molecular functions categories among *P. pectinatus* transcript clusters.

**Figure S8**. Log_2_-transformed TMM-normalized TPM of transcript clusters encoding gene products involved in nitrogen metabolism mapped to *Ca.* Sedimenticola endophacoides. Transcript clusters with zero TPM values are represented as white cells. Abbreviations: Nnr, nitrite and nitric oxide reductase; Nos, nitrous oxide reductase; Nor, nitric oxide reductase; Nap, periplasmic dissimilatory nitrate reductase.

**Figure S9**. Log_2_-transformed TMM-normalized TPM of transcript clusters encoding gene products involved in bacterial secretion systems mapped to *Ca.* Sedimenticola endophacoides. Transcript clusters with zero TPM values are represented as white cells. ‘*” indicates genes not binned in the species’ MAG. Abbreviations: Imp; inner membrane protein; VgrG, valine-glycine repeat protein G; Tag, type VI secretion-associated proteins; Tss, type VI secretion system proteins; Gsp, general secretory pathway protein; FtsY, signal recognition particle receptor; Ffh/SRP 54, subunit of the signal recognition particle; Sec, secretory export proteins; Tat, twin-arginine translocation proteins; PulE, pullulanase secretion protein E; ABC, ATP-binding cassette transporters; Tol, outer membrane proteins; DedD, cell division protein; DedA; conserved ancient membrane protein; HlyB, alpha-203 hemolysin translocation ATP-binding protein; HlyA, alpha-hemolysin; T1SS, type I secretion system; RTX, repeats in toxin; LapC, large adhesion protein (membrane fusion component).

# Supplemental References

Altschul SF, Gish W, Miller W, Myers EW, Lipman DJ (1990). Basic local alignment search tool. J Mol Biol 215:403-410, doi:10.1016/S0022-2836(05)80360-2.

Amann RI, Binder BJ, Olson RJ, Chisholm SW, Devereux R, Stahl DA (1990). Combination of 16S rRNA-targeted oligonucleotide probes with flow cytometry for analyzing mixed microbial populations. Appl Environ Microbiol 56:1919-1925.

Anisimova M, Gascuel O (2006). Approximate likelihood-ratio test for branches: a fast, accurate, and powerful alternative. Syst Biol 55:539-552, doi:10.1080/10635150600755453.

Babraham Bioinformatics (2010). FastQC - a quality control tool for high throughput sequence data. http://www.bioinformatics.babraham.ac.uk/projects/fastqc/.

Babraham Bioinformatics (2007). SeqMonk - A tool to visualise and analyse high throughput mapped sequence data. http://www.bioinformatics.babraham.ac.uk/projects/seqmonk/.

Benson DA, Clark K, Karsch-Mizrachi I, Lipman DJ, Ostell J, Sayers EW (2014). GenBank. Nucleic Acids Res, doi:10.1093/nar/gkt1030.

Bertrand EM, Saito MA, Jeon YJ, Neilan BA (2011). Vitamin B12 biosynthesis gene diversity in the Ross Sea: the identification of a new group of putative polar B12 biosynthesizers. Environ Microbiol 13:1285-1298, doi:10.1111/j.1462-2920.2011.02428.x.

Bowers RM, Kyrpides NC, Stepanauskas R, Harmon-Smith M, Doud D, Reddy TBK*, et al.* (2017). Minimum information about a single amplified genome (MISAG) and a metagenome-assembled genome (MIMAG) of bacteria and archaea. Nat Biotechnol 35:725-731, doi:10.1038/nbt.3893.

Brissac T, Mercot H, Gros O (2011). Lucinidae/sulfur-oxidizing bacteria: ancestral heritage or opportunistic association? Further insights from the Bohol Sea (the Philippines). FEMS Microbiol Ecol 75:63-76, doi:10.1111/j.1574-6941.2010.00989.x.

Davidson SK, Koropatnick TA, Kossmehl R, Sycuro L, McFall-Ngai MJ (2004). NO means ‘yes’ in the squid-vibrio symbiosis: nitric oxide (NO) during the initial stages of a beneficial association. Cell Microbiol 6:1139-1151, doi:10.1111/j.1462-5822.2004.00429.x.

Diepold A, Armitage JP (2015). Type III secretion systems: the bacterial flagellum and the injectisome. Philos Trans R Soc Lond B Biol Sci 370:20150020, doi:10.1098/rstb.2015.0020.

Doty TW (2015). Environmental controls on the diversity and distribution of endosymbionts associated with *Phacoides pectinatus* (Bivalvia: Lucinidae) from shallow mangrove and seagrass sediments, St. Lucie County, Florida. Master’s thesis (University of Tennessee, Knoxville, Tennessee, USA), http://trace.tennessee.edu/utk_gradthes/3548/.

Duperron S, Nadalig T, Caprais JC, Sibuet M, Fiala-Medioni A, Amann R*, et al.* (2005). Dual symbiosis in a *Bathymodiolus* sp. mussel from a methane seep on the Gabon continental margin (Southeast Atlantic): 16S rRNA phylogeny and distribution of the symbionts in gills. Appl Environ Microbiol 71:1694-1700, doi:10.1128/AEM.71.4.1694-1700.2005.

Duperron S, Fiala-Medioni A, Caprais J, Olu K, Sibuet M (2007). Evidence for chemoautotrophic symbiosis in a Mediterranean cold seep clam (Bivalvia: Lucinidae): comparative sequence analysis of bacterial 16S rRNA, APS reductase and RuBisCO genes. FEMS Microbiol Ecol 59:64-70, doi:10.1111/j.1574-6941.2006.00194.x.

Durand P, Gros O (1996). Bacterial host specificity of Lucinacea endosymbionts: interspecific variation in 16S rRNA sequences. FEMS Microbiol Lett 140:193-8, doi:10.1016/0378-1097(96)00178-4.

Edgar RC (2004). MUSCLE: a multiple sequence alignment method with reduced time and space complexity. BMC Bioinformatics 5:113, doi:10.1186/1471-2105-5-113.

Ehara T, Kitajima S, Kanzawa N, Tamiya T, Tsuchiya T (2002). Antimicrobial action of achacin is mediated by L-amino acid oxidase activity. FEBS Lett 531:509-512, doi:10.1016/S0014-5793(02)03608-6.

Good IJ (1953). The population frequencies of species and the estimation of population parameters. Biometrika 40:237-264.

Green-García AM, Engel AS (2012). Bacterial diversity of siliciclastic sediments in a *Thalassia testudinum* meadow and the implications for *Lucinisca nassula* chemosymbiosis. Estuar Coast Shelf Sci 112:153-161, doi:10.1016/j.ecss.2012.07.010.

Green-García AM (2008). Characterization of the lucinid bivalve-bacteria symbiotic system: The significance of the geochemical habitat on bacterial symbiont diversity and phylogeny. Master's thesis, (Louisiana State University, Baton Rouge, Louisiana, USA), https://digitalcommons.lsu.edu/gradschool_theses/1970/.

Guo C, Liu S, Yao Y, Zhang Q, Sun MZ (2012). Past decade study of snake venom L-amino acid oxidase. Toxicon 60:302-311, doi:10.1016/j.toxicon.2012.05.001.

Gurevich A, Saveliev V, Vyahhi N, Tesler G (2013). QUAST: quality assessment tool for genome assemblies. Bioinformatics 29:1072-1075, doi:10.1093/bioinformatics/btt086.

Haas BJ, Papanicolaou A, Yassour M, Grabherr M, Blood PD, Bowden J*, et al.* (2013). *De novo* transcript sequence reconstruction from RNA-Seq: Reference generation and analysis with Trinity. Nature protocols 8:1494-1512, doi:10.1038/nprot.2013.084.

Hall TA (1999). BioEdit: a user-friendly biological sequence alignment editor and analysis program for Windows 95/98/NT. Nucleic Acids Symp Ser 41:95-98.

Harris MA, Clark J, Ireland A, Lomax J, Ashburner M, Foulger R*, et al.* (2004). The Gene Ontology (GO) database and informatics resource. Nucleic Acids Res 32:D258-61, doi:10.1093/nar/gkh036.

Huson DH, Auch AF, Qi J, Schuster SC (2007). MEGAN analysis of metagenomic data. Genome Res 17:377-386, doi:10.1101/gr.5969107.

Joshie NA, Fass JN (2011). Sickle: a sliding-window, adaptive, quality-based trimming tool for FastQ files (Version 1.33). https://github.com/najoshi/sickle.

Kalmar Band Greensmith L (2009). Induction of heat shock proteins for protection against oxidative stress. Advanced Drug Delivery Reviews 61:310-318, doi:10.1016/j.addr.2009.02.003.

Kang DD, Froula J, Egan R, Wang Z (2015). MetaBAT, an efficient tool for accurately reconstructing single genomes from complex microbial communities. PeerJ 3:e1165, doi:10.7717/peerj.1165.

Kearse M, Moir R, Wilson A, Stones-Havas S, Cheung M, Sturrock S*, et al.* (2012). Geneious basic: an integrated and extendable desktop software platform for the organization and analysis of sequence data. Bioinformatics 28:1647-1649, doi:10.1093/bioinformatics/bts199.

Kimura M (1980). A simple method for estimating evolutionary rates of base substitutions through comparative studies of nucleotide sequences. J Mol Evol 16:111-120.

König S, Gros O, Heiden SE, Hinzke T, Thurmer A, Poehlein A*, et al.* (2016). Nitrogen fixation in a chemoautotrophic lucinid symbiosis. Nat Microbiol 2:16193, doi:10.1038/nmicrobiol.2016.193.

Konstantinidis KT, Tiedje JM (2005). Towards a genome-based taxonomy for prokaryotes. J Bacteriol 187:6258-6264, doi:10.1128/JB.187.18.6258-6264.2005.

Kozich JJ, Westcott SL, Baxter NT, Highlander SK, Schloss PD (2013). Development of a dual-index sequencing strategy and curation pipeline for analyzing amplicon sequence data on the MiSeq Illumina sequencing platform. Appl Environ Microbiol 79:5112-5120, doi:10.1128/AEM.01043-13.

Lane DJ (1991). 16S/23S rRNA sequencing. In: Nucleic Acid Techniques in Bacterial Systematics. John Wiley and Sons: New York, pp 115-175.

Langmead B, Salzberg SL (2012). Fast gapped-read alignment with Bowtie 2. Nature methods 9:357-359, doi:10.1038/nmeth.1923.

Le SQ, Gascuel O (2008). An improved general amino acid replacement matrix. Mol Biol Evol 25:1307-1320, doi:10.1093/molbev/msn067.

Li H, Handsaker B, Wysoker A, Fennell T, Ruan J, Homer N*, et al.* (2009). The sequence alignment/map format and SAMtools. Bioinformatics 25:2078-2079, doi:10.1093/bioinformatics/btp352.

Marcia M, Ermler U, Peng G, Michel H (2010). A new structure-based classification of sulfide:quinone oxidoreductases. Proteins 78:1073-1083, doi:10.1002/prot.22665.

Martin M (2011). Cutadapt removes adapter sequences from high-throughput sequencing reads. EMBnet journal 17:10-12, doi:10.14806/ej.17.1.200.

Moore S, Warren M (2012). The anaerobic biosynthesis of vitamin B_12_. Biochem Soc Trans 40:581-586, doi:10.1042/BST20120066.

Nakagawa S, Shimamura S, Takaki Y, Suzuki Y, Murakami S, Watanabe T*, et al.* (2014). Allying with armored snails: the complete genome of gammaproteobacterial endosymbiont. ISME J 8:40-51, doi:10.1038/ismej.2013.131.

NCBI Resource Coordinators (2016). Database resources of the National Center for Biotechnology Information. Nucleic Acids Res 44:D7-19, doi:10.1093/nar/gkv1290.

Nivaskumar M, Francetic O (2014). Type II secretion system: a magic beanstalk or a protein escalator. Biochim Biophys Acta 1843:1568-1577, doi:10.1016/j.bbamcr.2013.12.020.

Parks DH, Imelfort M, Skennerton CT, Hugenholtz P, Tyson GW (2015). CheckM: assessing the quality of microbial genomes recovered from isolates, single cells, and metagenomes. Genome Res 25:1043-1055, doi:10.1101/gr.186072.114.

Peters, JW, Schut, GJ, Boyd, ES, Mulder, DW, Shepard, EM, Broderick, JB, King, PW, Adams, MWW (2015). [FeFe]- and [NiFe]-hydrogenase diversity, mechanism, and maturation. BBA 1853: 1350-1369, doi: 10.1016/j.bbamcr.2014.11.021.

Petersen JM, Kemper A, Gruber-Vodicka H, Cardini U, van dG, Kleiner M*, et al.* (2016). Chemosynthetic symbionts of marine invertebrate animals are capable of nitrogen fixation. Nat Microbiol 2:16195, doi:10.1038/nmicrobiol.2016.195.

Ponnudurai R, Kleiner M, Sayavedra L, Petersen JM, Moche M, Otto A*, et al.* (2017). Metabolic and physiological interdependencies in the *Bathymodiolus azoricus* symbiosis. ISME J 11:463-477, doi:10.1038/ismej.2016.124.

Quast C, Pruesse E, Yilmaz P, Gerken J, Schweer T, Yarza P*, et al.* (2013). The SILVA ribosomal RNA gene database project: improved data processing and web-based tools. Nucleic Acids Res 41:D590-D596, doi:10.1093/nar/gks1219.

R Core Team (2016). R: a language and environment for statistical computing. https://www.r-project.org/.

Roux S, Enault F, Hurwitz BL, Sullivan MB (2015). VirSorter: mining viral signal from microbial genomic data. PeerJ 3:e985, doi:10.7717/peerj.985.

Schleicher TR, Nyholm SV (2011). Characterizing the host and symbiont proteomes in the association between the bobtail squid, *Euprymna scolopes*, and the bacterium, *Vibrio fischeri*. PloS one 6:e25649, doi:10.1371/journal.pone.0025649.

Schloss PD, Westcott SL, Ryabin T, Hall JR, Hartmann M, Hollister EB*, et al.* (2009). Introducing mothur: open-source, platform-independent, community-supported software for describing and comparing microbial communities. Appl Environ Microbiol 75:7537-7541, doi:10.1128/AEM.01541-09.

Seah B (2014). Phylogenomics-tools. doi: 10.5281/zenodo.46122. https://github.com/kbseah/phylogenomics-tools/.

Simao FA, Waterhouse RM, Ioannidis P, Kriventseva EV, Zdobnov EM (2015). BUSCO: assessing genome assembly and annotation completeness with single-copy orthologs. Bioinformatics 31:3210-3212, doi:10.1093/bioinformatics/btv351.

Small AL, McFall-Ngai MJ (1999). Halide peroxidase in tissues that interact with bacteria in the host squid *Euprymna scolopes*. J Cell Biochem 72:445-457, doi:10.1002/(SICI)1097-4644(19990315)72:43.0.CO;2-P.

Stamatakis A (2014). RAxML version 8: a tool for phylogenetic analysis and post-analysis of large phylogenies. Bioinformatics 30:1312-1313, doi:10.1093/bioinformatics/btu033.

Suzuki MT, Taylor LT, DeLong EF (2000). Quantitative analysis of small-subunit rRNA genes in mixed microbial populations via 5' nuclease assays. Appl Environ Microbiol 66:4605-4614, doi:10.1128/AEM.66.11.4605-4614.2000.

Takamatsu N, Shiba T, Muramoto K, Kamiya H (1995). Molecular cloning of the defense factor in the albumen gland of the sea hare *Aplysia kurodai*. FEBS Lett 377:373-376, doi:10.1016/0014-5793(95)01375-X.

Tamura K, Stecher G, Peterson D, Filipski A, Kumar S (2013). MEGA6: Molecular Evolutionary Genetics Analysis version 6.0. Mol Biol Evol 30:2725-2729, doi:10.1093/molbev/mst197.

The UniProt Consortium (2015). UniProt: a hub for protein information. Nucleic Acids Res 43:D204-D212, doi:10.1093/nar/gku989.

Thompson JD, Higgins DG, Gibson TJ (1994). CLUSTAL W: improving the sensitivity of progressive multiple sequence alignment through sequence weighting, position-specific gap penalties and weight matrix choice. Nucleic Acids Res 22:4673-4680.

Tomich M, Planet PJ, Figurski DH (2007). The *tad* locus: postcards from the widespread colonization island. Nat Rev Microbiol 5:363-375, doi:10.1038/nrmicro1636.

Turner S, Pryer KM, Miao VP, Palmer JD (1999). Investigating deep phylogenetic relationships among cyanobacteria and plastids by small subunit rRNA sequence analysis. J Eukaryot Microbiol 46:327-338, doi:10.1111/j.1550-7408.1999.tb04612.x.

Untergasser A, Cutcutache I, Koressaar T, Ye J, Faircloth BC, Remm M*, et al.* (2012). Primer3 - new capabilities and interfaces. Nucleic Acids Res 40:e115, doi:10.1093/nar/gks596.

Varghese NJ, Mukherjee S, Ivanova N, Konstantinidis KT, Mavrommatis K, Kyrpides NC*, et al.* (2015). Microbial species delineation using whole genome sequences. Nucleic Acids Res 43:6761-6771, doi:10.1093/nar/gkv657.

Wallner G, Amann R, Beisker W (1993). Optimizing fluorescent in situ hybridization with rRNA-targeted oligonucleotide probes for flow cytometric identification of microorganisms. Cytometry 14:136-143, doi:10.1002/cyto.990140205.

Weis VM, Small AL, McFall-Ngai MJ (1996). A peroxidase related to the mammalian antimicrobial protein myeloperoxidase in the *Euprymna*-*Vibrio* mutualism. Proc Natl Acad Sci U S A 93:13683-13688.

Wu M, Eisen JA (2008). A simple, fast, and accurate method of phylogenomic inference. Genome Biol 9:R151, doi:10.1186/gb-2008-9-10-r151.

Xi H, Schneider BL, Reitzer L (2000). Purine catabolism in *Escherichia coli* and function of xanthine dehydrogenase in purine salvage. J Bacteriol 182:5332-5341.
